# *GmPHR25*, a *GmPHR* member up-regulated by phosphate starvation, controls phosphate homeostasis in soybean

**DOI:** 10.1093/jxb/erx292

**Published:** 2017-08-23

**Authors:** Ying-Bin Xue, Bi-Xian Xiao, Sheng-Nan Zhu, Xiao-Hui Mo, Cui-Yue Liang, Jiang Tian, Hong Liao

**Affiliations:** 1 Root Biology Center, State Key Laboratory for Conservation and Utilization of Subtropical Agro-bioresources, South China Agricultural University, Guangzhou, P.R. China; 2 Root Biology Center, Haixia Institute of Science and Technology, Fujian Agriculture and Forestry University, Fuzhou, P.R. China

**Keywords:** Expression pattern, *PHR*, Pi homeostasis, Pi starvation, soybean

## Abstract

As an essential nutrient element, phosphorus (P) plays an important role in plant growth and development. Low P availability is a limiting factor for crop production, especially for legume crops (e.g. soybean), which require additional P to sustain nitrogen fixation through symbiotic associations with rhizobia. Although PHOSPHATE STARVATION RESPONSE 1 (PHR1) or PHR1-like is considered as a central regulator of phosphate (Pi) homeostasis in several plant species, it remains undefined in soybean. In this study, 35 *GmPHR* members were cloned from the soybean genome and expression patterns in soybean were assayed under nitrogen (N) and P deficiency conditions. *GmPHR25*, which is up-regulated in response to Pi starvation, was then overexpressed in soybean hairy roots *in vitro* and *in vivo* to investigate its functions. The results showed that overexpressing *GmPHR25* increased Pi concentration in transgenic soybean hairy roots under normal conditions, accompanied with a significant decrease in hairy root growth. Furthermore, transcripts of 11 out of 14 high-affinity Pi transporter (*GmPT*) members as well as five other Pi starvation-responsive genes were significantly increased in soybean hairy roots with *GmPHR25* overexpression. Taken together, this study suggests that *GmPHR25* is a vital regulator in the P signaling network, and controls Pi homeostasis in soybean.

## Teaser

*GmPHR25*, which is up-regulated by phosphate starvation, is a vital regulator in the phosphorus signaling network, and controls phosphate homeostasis in soybean.

## Introduction

Phosphorus (P) is an essential macronutrient in plants that is not only a major constituent in plant cells, but is also involved in metabolic processes (Raghothama, 1999; Richardson, 2009). Furthermore, meeting crop P needs with supplemental additions is problematic due to the fact that applied phosphate (Pi) fertilizers are easily fixed by soil particles into unavailable forms (e.g. aluminum, iron, and calcium phosphates), which results in low soil Pi availability (Beardsley, 2011; Veneklaas *et al.*, 2012). Equally concerning is the prediction that the rock phosphate sources used in fertilizers will be largely depleted within a number of decades (Vance *et al.*, 2003; Cordell *et al.*, 2009). Therefore, development of ‘smart’ crop cultivars with superior P-use efficiency and optimization of field P management are imperative for the future of sustainable agriculture (Shen *et al.*, 2011; Tian *et al.*, 2012; Veneklaas *et al.*, 2012; Wu *et al.*, 2013).

Plants have evolved a range of morphological, physiological, and molecular strategies in adaptation to P deficiency, including changes of root morphology and architecture, increased exudation of organic acids and purple acid phosphatases, and formation of symbiotic interactions with arbuscular mycorrhizal (AM) fungi (Veneklaas *et al.*, 2012). Many of these adaptive strategies enhance soil P mobility or plant acquisition of this limiting resource, and thus increase P efficiency. In recent years, identification of the multiple genes and proteins that regulate the relevant adaptive processes has significantly contributed to an emerging picture of the complex signaling network involved in plant responses to P deficiency.

Among the genes and proteins identified, several are considered as vital regulators, including a plant small ubiquitin-like modifier E3 ligase (SIZ1), *PHR1*, *microRNA399*, and proteins containing the SYG1/PHO81/XPR1 (SPX) domain (Chiou and Lin, 2011; Veneklaas *et al.*, 2012; Wu *et al.*, 2013; Liang *et al.*, 2014). As a MYB-CC type transcription factor, *PHR1* and its homologs appear to play central roles in P signaling networks (Chiou and Lin, 2011; Liang *et al.*, 2013; Sun *et al.*, 2016). *Phosphate Starvation Response 1* (*CrPSR1*) is an ancestral MYB-CC type transcription factor that may be critical for the acclimation of the unicellular green alga *Chlamydomonas reinhardtii* to P deficiency (Wykoff *et al.*, 1999). In plants, the identification and functional analysis of the *CrPSR1* homolog *AtPHR1* was a milestone accomplishment along the path to elucidating the P signaling network in Arabidopsis (*Arabidopsis thaliana*) (Rubio *et al.*, 2001). Over time, *phr1* mutations have been associated with decreases in anthocyanin accumulation and Pi concentration, as well as lower root-to-shoot ratios (Rubio *et al.*, 2001; Bustos *et al.*, 2010). Furthermore, transcription of multiple Pi starvation-responsive genes is impaired in *phr1* mutants (Rubio *et al.*, 2001; Bustos *et al.*, 2010). At the opposite extreme, overexpression of *AtPHR1* in Arabidopsis leads to significant increases in Pi concentration, accompanied by increased transcription of Pi starvation-responsive genes, such as *miR399*, *RNase 1*, and *PHOSPHATE TRANSPORTER 1–7* (Nilsson *et al.*, 2007). One of these Pi starvation-responsive genes, *miR399*, is suggested to repress expression levels of a *ubiquitin-conjugating E2*, *PHO2*, which regulates the abundance of PHOSPHATE TRANSPORTER (PHT), and thereby modulates Pi acquisition and accumulation (Fujii *et al.*, 2005). Thus, it is well known that *PHR1*, *miR399*, *PHO2*, and *PHT* form a branch of the P signaling network controlling Pi homeostasis in plants (Bari *et al.*, 2006; Chiou and Lin, 2011; Liang *et al.*, 2014).

Recently, *PHR1* homologs have also been documented to play important roles in regulating Pi homeostasis, including *PHL1* and *PHL2* in Arabidopsis, *OsPHR1*, *OsPHR2*, *OsPHR3*, and *OsPHR4* in rice (*Oryza sativa*), *BnPHR1* in rape (*Brassica napus*), *TaPHR1* in wheat (*Triticum aestivum*), *ZmPHR1* in maize (*Zea mays*), and *PvPHR1* in bean (*Phaseolus vulgaris*) (Valdés-lópez *et al.*, 2008; Zhou *et al.*, 2008; Bustos *et al.*, 2010; Ren *et al.*, 2012; Wang et al., 2013*a*, 2013*b*; Guo *et al.*, 2015; Sun *et al.*, 2015; Ruan *et al.*, 2017). Furthermore, binding of AtPHR1 and OsPHR2 to the P1BS site (PHR1-binding sequence: GNATATNC) is inhibited by Pi-dependent interactions with AtSPX1 and AtSPX2 in Arabidopsis and with OsSPX1, OsSPX2, and OsSPX4 in rice, which suggests the presence of another layer of complexity in the P signaling network in plants (Lv *et al.*, 2014; Puga *et al.*, 2014; Wang *et al.*, 2014; Zhou *et al.*, 2015).

Although much of the complex P signaling network has been elucidated in model plants (e.g. rice and Arabidopsis), genome-wide analysis of *PHR* members responsive to Pi starvation and their functions in controlling Pi homeostasis remain scarce and fragmentary in other plants, notably in legume crops. Soybean (*Glycine max*) is an important oil-bearing legume with high nutritional value (Herridge *et al.*, 2008). It has been demonstrated that soybean exhibits multiple adaptive strategies to P deficiency, including formation of a shallower root system, increases of organic exudation and acid phosphatase (APase) activity, and alterations in symbiotic associations with AM fungi and rhizobia (Tian *et al.*, 2003; Zhao *et al.*, 2004; Liao *et al.*, 2006; Liu *et al.*, 2008; Qin *et al.*, 2011; Liang *et al.*, 2013).

With the availability of soybean genome sequences, expression analysis for soybean responses to Pi starvation has been conducted for several gene families, including *expansin* (*EXPB*), *purple acid phosphatase* (*PAP*), *phosphate transporter* (*PT*), and *SPX* (Wu *et al.*, 2011; Li *et al.*, 2012; Qin *et al.*, 2012*a*; Fan *et al.*, 2013; Li *et al.*, 2014; Yao *et al.*, 2014). Furthermore, functional analysis of several Pi starvation-responsive genes has led to elucidation of the molecular mechanisms underlying soybean adaptations to P deficiency. For example, a *β-expansin* gene, *GmEXPB2*, is highly induced in soybean roots by P deficiency, and overexpressing *GmEXPB2* in Arabidopsis leads to enhanced root growth and Pi uptake (Guo *et al.*, 2011). In addition, *GmPT5*, a high-affinity phosphate transporter, is mainly expressed in nodules and plays an important role in maintenance of Pi homeostasis in soybean nodules (Qin *et al.*, 2012*b*). Furthermore, *GmSPX3* has recently been suggested as a critical regulator in the P signaling network because it regulates transcription of a group of Pi starvation-responsive genes in soybean, including *GmEXPB2* and *GmPT5* (Yao *et al.*, 2014).

Despite this progress in elucidating P signaling networks in soybean, the role of *GmPHR* members in these networks remains unclear. In the present study, genome-wide analysis of 35 *GmPHR* members was conducted. Beyond identification and phylogenetic analysis, expression patterns of *GmPHR* members were examined in different soybean tissues in response to P deficiency. Furthermore, functional analysis of a *GmPHR* member up-regulated by Pi starvation, *GmPHR25*, suggests that it is a key regulator in the P signaling network controlling Pi homeostasis in soybean.

## Materials and methods

### Identification of the *GmPHR* family in soybean

BLAST searches were performed, firstly using the *AtPHR1* (AT4G28610) sequence as a query sequence. Then, using all identified *GmPHR* sequences as query sequences at the phytozome website (http://www.phytozome.net), a total of 35 *GmPHR* members were identified in the soybean genome that harbor two conserved domains (i.e. MYB and Coiled-Coil) and exhibit more than 24% similarity with *AtPHR1*. The members of the *GmPHR* family were named *GmPHR1* to *GmPHR35* based on their positions on the chromosomes. General information for each *GmPHR* member (e.g. numbers of exons and introns, length of open reading frame) was extracted from the same website. Protein molecular weights were predicted using the ExPASy web server (http://www.expasy.org/). Phylogenetic tree analysis of the PHR proteins was conducted using a ClustalX multiple-sequence alignment and the neighbor-joining method with 1000 bootstrap replicates in MEGA 5.05, as described previously (Tamura *et al.*, 2007).

### Plant growth conditions

The soybean genotype YC03-3 was used in these experiments. For expression analysis of *GmPHR* members in various soybean tissues, from 7 d after seed germination seedlings were grown in a full-strength nutrient solution containing 1500 μM KNO_3_, 1200 μM Ca(NO_3_)_2_, 400 μM NH_4_NO_3_, 500 μM KH_2_PO_4_, 500 μM MgSO_4_, 25 μM MgCl_2_, 300 μM K_2_SO_4_, 300 μM (NH_4_)_2_SO_4_, 1.5 μM MnSO_4_, 1.5 μM ZnSO_4_, 0.5 μM CuSO_4_, 0.16 μM (NH_4_)_6_Mo_7_O_24_, 2.5 μM NaB_4_O_7_, and 40 μM Fe-EDTA. On day 25 of growth in the nutrient solution, entirely expanded young leaves, roots, and flowers were harvested separately. Pods of 1 cm length and immature seeds were harvested separately on days 30 and 40 after transferring seedlings into the nutrient solution. All samples were stored at −80 °C prior to RNA extraction.

For the nutrient deficiency experiment, 7 d after seed germination, soybean seedlings were grown for 14 d in complete nutrient solution as a control, or in nutrient solution without nitrogen (N) or phosphorus (P). Then, entirely expanded young leaves and roots were harvested separately for further analysis, as described previously (Yao *et al.*, 2014). Briefly, in the N deficiency (–N) solution, K_2_SO_4_ and CaCl_2_ were used to replace the KNO_3_ and Ca(NO_3_)_2_, respectively. In the P deficiency (–P) solution, K_2_SO_4_ replaced KH_2_PO_4_. For the rhizobium inoculation experiment, 7 d after seed germination, soybean seedlings were inoculated with rhizobium, *Bradyrhizobium* sp. BXYD3, for 1 h, and then transferred into low-nitrogen (100 μM total N) nutrient solution containing 5 μM or 500 μM KH_2_PO_4_, as described previously (Yao *et al.*, 2014). Nodules were harvested at 30 d after inoculation for further analysis. All experiments included four biological replicates.

### RNA extraction and quantitative real-time PCR analysis

Total RNA was extracted from various soybean tissues using TRIzol reagent (Invitrogen, USA) according to the manufacturer’s instructions. Subsequently, RNA samples were treated with RNase-free *DNase* I (TaKaRa, Japan) to remove genomic DNA. The first cDNA strand was synthesized using MMLV-reverse transcriptase (Promega, USA) according to the given protocol. Quantitative real-time PCR (qPCR) was performed and analysed using SYBR Green PCR master mix (Promega, USA) and a Rotor-Gene 3000 system (Corbett Research, Australia). Expression levels of the soybean housekeeping gene, *EF1-α* (Glyma.17G186600) or *ACTIN* (Glyma.18G290800) were used as an endogenous control to normalize the samples, as described previously (Li *et al.*, 2015; Liu *et al.*, 2016). The specific primer sequences used in the study are listed in Supplementary Data Table S2 at *JXB* online.

### Subcellular localization of GmPHR25

To determine the subcellular localization of GmPHR25, the coding region of *GmPHR25* was amplified with specific primers (Supplementary Table S3), and cloned into the *pMDC43* vector and the *pBEGFP* vector for fusion with green fluorescent protein (GFP) at its N- and C-terminus, respectively, according to the manufacturer’s instructions (Invitrogen, USA). The plasma membrane marker *AtPIP2A-mCherry* was used for co-localization analysis. Each of *35S:GFP*, *35S:GFP-GmPHR25*, *35S:GmPHR25-GFP*, and *35S:AtPIP2A-mCherry* fusion vectors was separately introduced into *Agrobacterium tumefaciens* strain GV3101, and then transformed into tobacco (*Nicotiana benthamiana*) leaves for transient expression, as described previously (Liu *et al.*, 2012). After 3 d, transformed tobacco leaf epidermal cells were imaged on a Zeiss LSM7 DUO confocal microscope (Zeiss, Germany). Fluorescence of GFP and mCherry was stimulated at 488 and 543 nm, respectively.

### *In vitro* overexpression of *GmPHR25* in soybean hairy roots

The coding region of *GmPHR25* was amplified with specific primers (Supplementary Table S3), and the PCR product was ligated into the *pYLRNAi* vector after digestion by *Sac* I and *Pst* I. *GmPHR25*-OE or empty vector constructs were separately transformed into *Agrobacterium rhizogenes* strain K599, which was further used to infect soybean cotyledons to obtain transgenic hairy roots *in vitro*, as described previously (Guo *et al.*, 2011). Transgenic hairy roots were grown on Murashige and Skoog (MS) medium supplied with carbenicillin, and were then confirmed by PCR and qPCR analysis. Two independent *GmPHR25* overexpression lines and the control line were selected, and established for further experiments. About 0.2 g (fresh weight) of hairy roots from each of the three independent lines was sub-cultured in MS medium supplied with 1.25 mM KH_2_PO_4_ (+P) or without KH_2_PO_4_ (–P). After 14 d of growth, hairy roots were harvested for dry weight and Pi concentration analysis, as described previously (Liang *et al.*, 2010). Each independent transgenic line had three biological replicates.

### *GmPHR25* overexpression and suppression in soybean composite plants

To construct the *GmPHR25*-RNAi vector, a 360-bp specific fragment from the *GmPHR25* coding region was amplified with specific primers (Supplementary Table S3), and PCR products were ligated into the *pYLRNAi* vector after digestion by *BamH* I and *Hind* III, *Mlu* I and *Pst* I. Subsequently, *GmPHR25*-OE, *GmPHR25*-RNAi, or empty vector constructs were separately transformed into *Agrobacterium rhizogenes* strain K599, which was also used to infect soybean seedlings in order to obtain composite soybean plants with transgenic hairy roots, as described previously (Guo *et al.*, 2011). When transgenic hairy roots grew to approximately 10 cm long, a small portion was harvested for PCR and qPCR analysis. The transgenic composite soybean plants were grown in nutrient solution supplied with 500 µM KH_2_PO_4_ (+P) or 25 µM KH_2_PO_4_ (–P). For each P treatment, six independent transgenic lines were included for *GmPHR25*-OE, *GmPHR25*-RNAi, or control lines. After 14 d of growth, entirely expanded young leaves, shoots, and hairy roots were separately harvested to determine dry weight, along with total P and soluble Pi concentration, as described previously (Liang *et al.*, 2010). Small portions of hairy roots were also harvested for further qPCR analysis. One independent transgenic line originating from a composite soybean plant with transgenic hairy roots was considered as a semi-biological replicate. A total of six replicates were included in this experiment.

### Measurement of soluble Pi and total P concentration

For the soluble Pi concentration assay, about 0.1 g samples of fresh plant tissue were ground and extracted by distilled water. After centrifugation, the supernatant was assayed as described previously (Murphy and Riley, 1962). For the plant total P concentration assay, whole plants were heated at 75 °C until completely dry, then shoots and roots were ground into powder and, after digestion by H_2_SO_4_, Pi concentration was determined as above.

### Expression analysis of downstream genes in soybean composite plants

Total RNA was extracted from transgenic hairy roots in soybean composite plants, and then qPCR was conducted to analyse transcription of downstream genes, including *GmHAD1-2* (Glyma07g01410), *GmSPX5* (Glyma10g40820), *GmEXPB2* (Glyma10g24080), *GmPAP14* (Glyma08g09880), *GmPAP21* (Glyma10g08300), 14 soybean *high-affinity phosphate transporter* (*GmPT*) members (Qin *et al.*, 2012*a*), and the other 34 *GmPHR* members (i.e. except *GmPHR25*). All qPCR primers were designed according to sequences from the phytozome website (http://www.phytozome.net).

### Transcriptional activity and DNA-binding affinity analysis of GmPHR25

To detect transcriptional activity of GmPHR25, the full-length *GmPHR25* coding region was amplified with specific primers (Supplementary Table S3) and inserted into *pGBKT7* fused with *GAL4 DNA-BD* using the Matchmaker yeast two-hybrid system (Clontech, USA). The constructs were transformed into yeast strain AH109, and screened on the minimal medium SD/-Trp and SD/-Trp-His-A to examine the reporter gene expression. Yeast transformed with the empty *pGBKT7* (*BD*) vector was used as a negative control.

For detection of DNA-binding affinity of GmPHR25, three soybean high-affinity Pi transporters (*GmPT9*, *GmPT10*, and *GmPT12*) were selected because their expression levels were up-regulated by *GmPHR25* and their promoter region contains one or two PHR1 biding sites (P1BS; 5′-GNATATNC-3′). Therefore, fragments were separately amplified from the promoter regions of *GmPT9*, *GmPT10*, and *GmPT12* using specific primers containing at least one P1BS element (Supplementary Table S3), which were subsequently cloned into *pABAi* vectors. The constructs were transformed into yeast strain Y1HGold (Clontech, USA). Meanwhile, a fragment containing four P1BS (5′-GAATATTC-3′) elements was synthesized and cloned into the *pABAi* vector as the positive control, as described previously (Sun *et al.*, 2016; Ruan *et al.*, 2017). The full-length *GmPHR25* coding region was amplified and cloned into the *pGADT7* (*AD*) vector and transformed into yeast bait strain. Yeast transformed with *pGADT7* was used as a negative control. Modified medium without uracil (Ura) or leucine (Leu) was used for selection.

### Statistical analyses

All data were analysed using Microsoft Excel 2003 (Microsoft Company, USA) to calculate means and standard errors, and SPSS 10.1 (SPSS Institute Inc., Cary, NC, USA) was used to conduct Student’s *t*-tests.

## Results

### Identification and characterization of *GmPHR* members in soybean

A total of 35 putative *GmPHR* members were identified through BLAST searching of the soybean genome database at http://www.phytozome.net, And general information on them is summarized in Table 1. The *GmPHR* members were unevenly distributed on soybean chromosomes 1–3, 7–13, 15, 16, and 18–20 (Table 1). Based on their positions on these chromosomes, the 35 *GmPHR* members were named from *GmPHR1* to *GmPHR35*. As shown in Table 1, open reading frames of the *GmPHR* members ranged from 642 to 1455 bp in length, which was predicted to encode proteins containing 213–484 amino acids, and exhibiting 41–61% sequence identity with AtPHR1 (Table 1).

**Table 1.** General information for the 35 *GmPHR* members

Gene	Locus	Chromosomal location	Exon/Intron number	Length of ORF (bp)	Number of amino acids (aa)	Protein size (kD)	Identity to AtPHR1
*GmPHR1*	Glyma01g01300	1	6/5	813	270	30.3	48%
*GmPHR2*	Glyma01g05920	1	6/5	1035	334	38.2	45%
*GmPHR3*	Glyma02g07790	2	6/5	1251	416	46.9	46%
*GmPHR4*	Glyma02g12070	2	6/5	1056	351	39.2	44%
*GmPHR5*	Glyma02g30714	2	7/6	642	213	24.3	48%
*GmPHR6*	Glyma02g30800	2	6/5	1269	422	47.1	54%
*GmPHR7*	Glyma03g00590	3	6/5	804	267	29.0	58%
*GmPHR8*	Glyma03g29940	3	6/5	1284	427	47.7	58%
*GmPHR9*	Glyma03g32350	3	7/6	1446	481	53.2	50%
*GmPHR10*	Glyma03g41040	3	8/7	1308	435	48.6	43%
*GmPHR11*	Glyma07g35700	7	6/5	996	331	37.3	43%
*GmPHR12*	Glyma08g17400	8	6/5	1122	373	41.4	42%
*GmPHR13*	Glyma09g02030	9	6/5	945	314	34.3	42%
*GmPHR14*	Glyma09g02040	9	6/5	990	329	35.7	53%
*GmPHR15*	Glyma09g17404	9	6/5	1275	424	47.0	61%
*GmPHR16*	Glyma09g34461	9	6/5	798	265	30.0	48%
*GmPHR17*	Glyma10g04540	10	7/6	1446	481	53.0	54%
*GmPHR18*	Glyma10g34050	10	6/5	1164	387	43.1	41%
*GmPHR19*	Glyma11g18990	11	6/5	1245	414	46.2	44%
*GmPHR20*	Glyma12g09490	12	6/5	1218	405	45.2	45%
*GmPHR21*	Glyma12g31020	12	6/5	1263	420	47.4	47%
*GmPHR22*	Glyma13g18805	13	7/6	1440	479	53.0	54%
*GmPHR23*	Glyma13g39290	13	6/5	1203	400	45.0	47%
*GmPHR24*	Glyma15g12930	15	6/5	942	313	34.2	42%
*GmPHR25*	Glyma15g12940	15	6/5	990	329	35.6	54%
*GmPHR26*	Glyma15g29620	15	6/5	1068	355	39.6	49%
*GmPHR27*	Glyma15g41740	15	7/6	1164	387	43.2	45%
*GmPHR28*	Glyma16g26820	16	6/5	1251	416	46.9	47%
*GmPHR29*	Glyma18g43130	18	6/5	714	237	26.8	47%
*GmPHR30*	Glyma19g30220	19	6/5	819	272	29.6	58%
*GmPHR31*	Glyma19g32850	19	6/5	1206	401	45.2	52%
*GmPHR32*	Glyma19g35080	19	7/6	1455	484	53.3	50%
*GmPHR33*	Glyma19g43690	19	8/7	1209	402	44.7	44%
*GmPHR34*	Glyma20g04630	20	6/5	1005	334	37.4	43%
*GmPHR35*	Glyma20g33540	20	6/5	1182	393	43.2	42%

Gene locus, exon and intron number, length, and protein size were extracted from the Phytozome website (http://www.phytozome.net). The identity between AtPHR1 and each GmPHR member was determined by BLAST analysis (https://blast.ncbi.nlm.nih.gov/Blast.cgi). ORF, open reading frame.

**Fig. 1.**
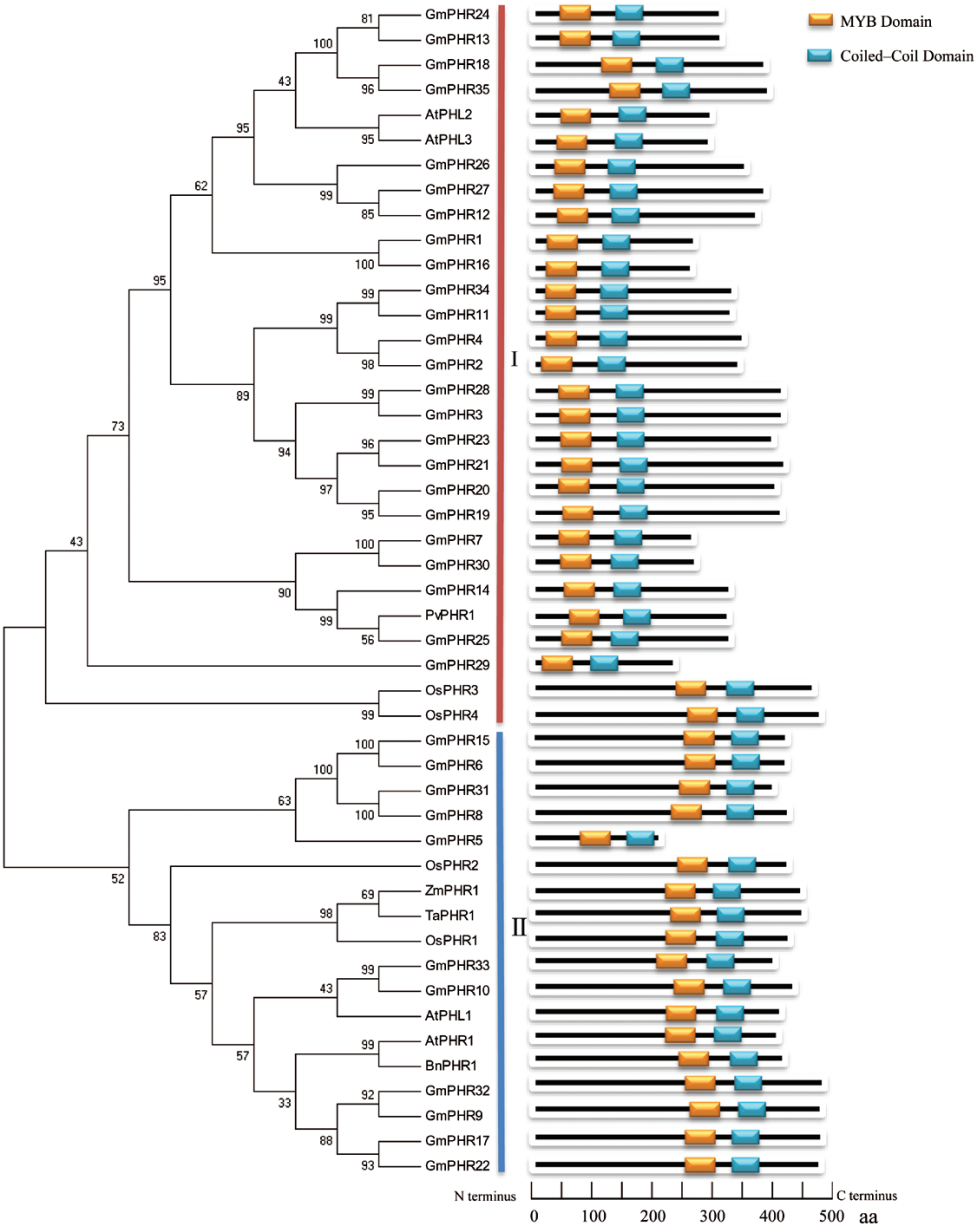
Phylogenetic analysis of PHR proteins. For information on the soybean proteins see Table 1. The GenBank accession numbers of the proteins or gene loci for other species are as follows: AtPHR1 (At4g28610), AtPHL1 (At5g29000), AtPHL2 (At3g24120), AtPHL3 (At4g13640), OsPHR1 (Os03g21240), OsPHR2 (Os07g25710), OsPHR3 (Os02g04640), OsPHR4 (Os06g49040), BnPHR1 (JN806156), TaPHR1 (KC218925), ZmPHR1 (JF831533), PvPHR1 (EU500763). At, *Arabidopsis thaliana*; Gm, *Glycine max*; Os, *Oryza sativa*; Pv, *Phaseolus vulgaris*; Bn, *Brassica napus*; Zm, *Zea mays*; Ta, *Triticum aestivum*. The phylogenetic tree was created using the Mega 5.05 program.

In order to determine evolutionary relationships among PHR members in soybean, Arabidopsis, rice, bean, wheat, maize, and rape, a phylogenetic tree was constructed. The results showed that plant PHR proteins can be divided into two groups, labeled as group I and group II in Fig. 1. Group I consisted of 24 GmPHR members together with AtPHL2 and AtPHL3 from Arabidopsis, PvPHR1 from bean, OsPHR3 and OsPHR4 from rice; however, 11 other GmPHR members were classified into group II, including GmPHR5, 6, 8, 9, 10, 15, 17, 22, 31, 32, 33, together with AtPHR1 and AtPHL1 from Arabidopsis, OsPHR1 and OsPHR2 from rice, ZmPHR1 from maize, TaPHR1 from wheat, and BnPHR1 from rape, (Fig. 1). Furthermore, two typical domains of PHR members, MYB binding and Coiled-Coil, were closely localized on the C-terminus for all PHR members in group II, but on the N-terminus for all PHR members in group I, except for OsPHR3 and OsPHR4 (Fig. 1).

### Tissue-specific expression of *GmPHR* members

**Fig. 2.**
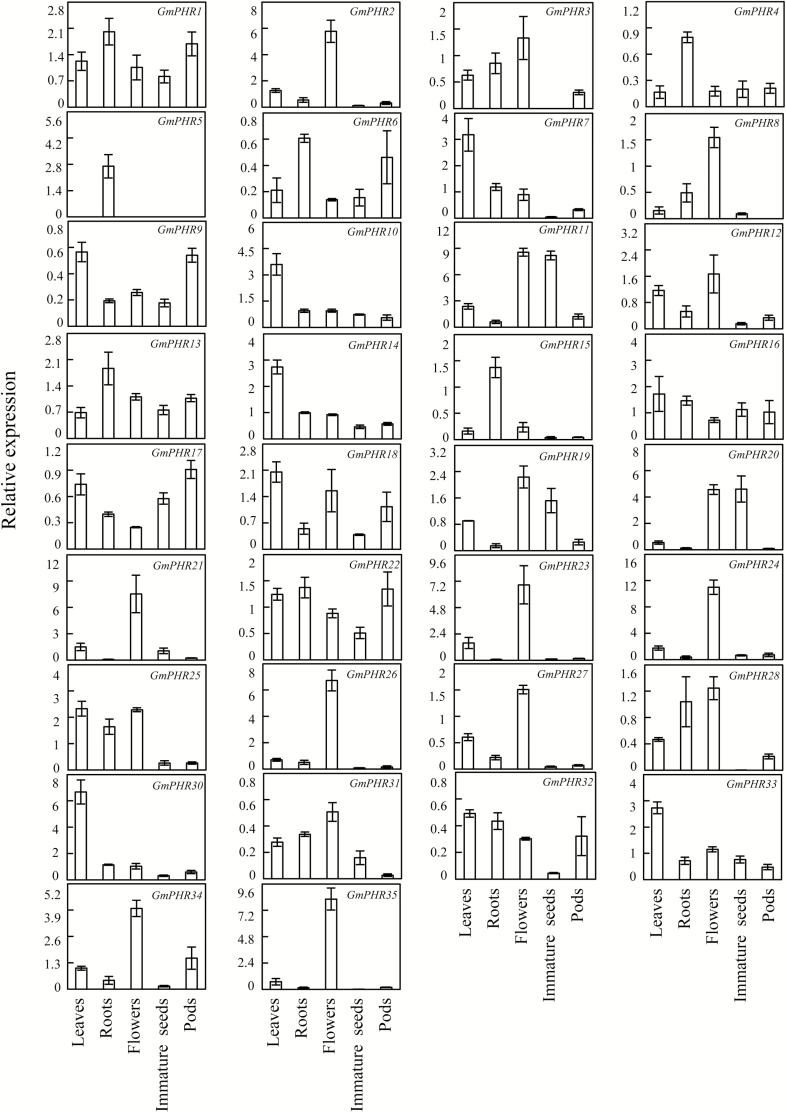
Tissue-specific expression patterns of *GmPHR* members. Soybean seedlings were grown in complete nutrient solution and entirely expanded young leaves, roots, flowers, 1-cm pods, and immature seeds were separately harvested for qPCR analysis. Data are means of four replicates ±SE.

Expression patterns of *GmPHR* members were determined by qPCR analysis of soybean leaves, roots, flowers, pods, and seeds. The results showed that transcripts could be detected for all *GmPHR* members except for *GmPHR29* (Fig. 2), and that expression patterns varied throughout the tissues that were tested . For example, *GmPHR7*, *GmPHR10*, *GmPHR14*, *GmPHR30*, and *GmPHR33* were most highly expressed in leaves, *GmPHR2*, *GmPHR8*, *GmPHR21*, *GmPHR23*, *GmPHR24*, *GmPHR26*, *GmPHR27*, *GmPHR34*, and *GmPHR35* were most highly expressed in flowers, and *GmPHR4*, *GmPHR5*, and *GmPHR15* were mainly expressed in roots. The expressions of *GmPHR11*, *GmPHR19*, and *GmPHR20* were higher in flowers and immature seeds than in other tissues. For *GmPHR32*, expression levels were similar in leaves and other tissues, except for immature seeds (Fig. 2).

### Transcriptional responses of *GmPHR* to nutrient deficiencies

**Fig. 3.**
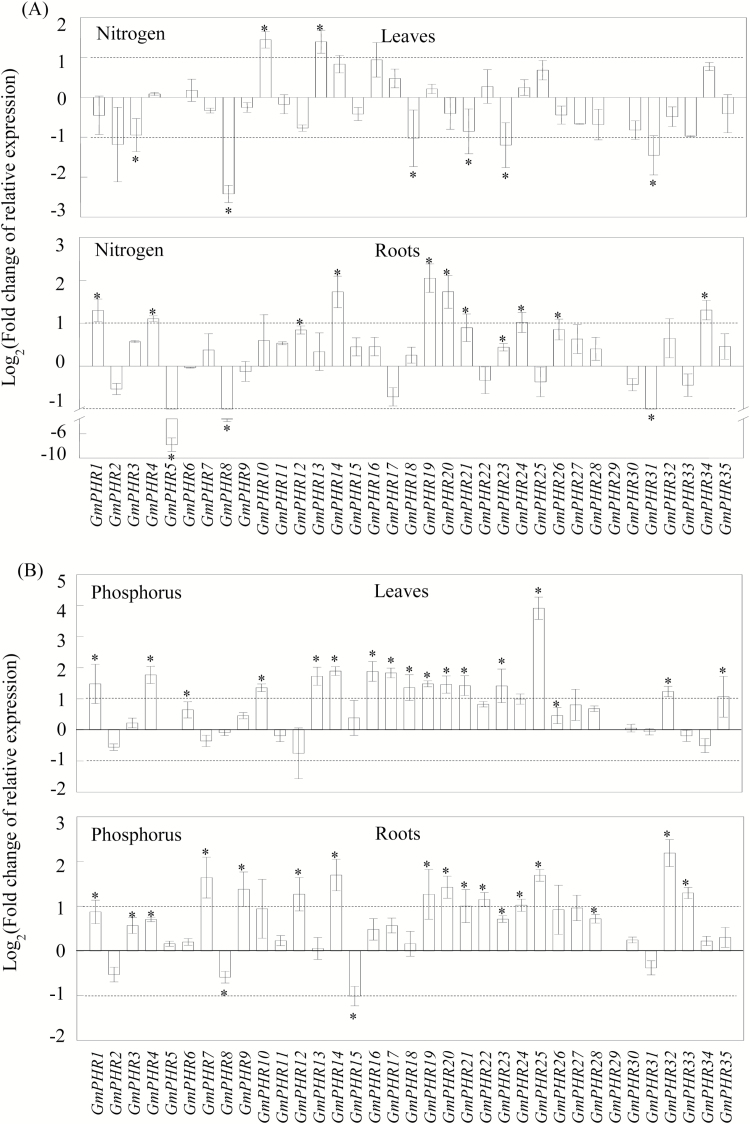
Expression patterns of *GmPHR* members in response to nitrogen and phosphorus deficiency in leaves and roots. Soybean seedlings were grown for 14 d in complete nutrient solution as a control, or in nutrient solution without nitrogen (A) or phosphorus (B). Data are means of four replicates ±SE, expressed as the binary logarithm of fold-changes of relative expression of *GmPHR* members under nutrient deficiency compared with normal conditions. *, Significant difference between normal and nutrient deficiency treatments (Student’s *t*-test, *P*<0.05).

Expression patterns of *GmPHR* members in both leaves and roots were further examined under nitrogen (N) or phosphorus (P) nutrient deficiency conditions. The results showed that *GmPHR* members exhibited diverse responses to N and P deficiencies (Fig. 3).

In the N deficiency treatment, the transcription of most *GmPHR* members remained largely unchanged, with fold-changes below 2 compared with controls observed in both soybean leaves and roots for all genes, except for six members in leaves and ten members in roots (Fig. 3A). In leaves, only *GmPHR10* and *GmPHR13* were found to be significantly up-regulated by more than 2-fold, while *GmPHR8*, *GmPHR18*, *GmPHR23*, and *GmPHR31* were significantly down-regulated by more than 2-fold by nitrogen starvation (Fig. 3A). In roots, the expressions of seven *GmPHR* members (*GmPHR1*, *4*, *14*, *19*, *20*, *24*, *34*) were significantly up-regulated by more than 2-fold under nitrogen deficiency conditions, while the expressions of *GmPHR5*, *GmPHR8*, and *GmPHR31* were significantly down-regulated by more than 2-fold (Fig. 3A).

In contrast to N deficiency, expression levels of most *GmPHR* members were significantly up-regulated by Pi starvation in soybean leaves and roots (Fig. 3B). In leaves, transcription of 15 members (*GmPHR1*, *4*, *10*, *13*, *14*, *16–21*, *23*, *25*, *32, 35*) was significantly increased by more than 2-fold in the P deficiency treatment, especially for *GmPHR25*, which exhibited a 16-fold increase of transcript levels in response to P deficiency (Fig. 3B). Similarly, in roots, except for *GmPHR15* where expression was significantly down-regulated by more than 2-fold, expression levels were significantly up-regulated by more than 2-fold by Pi starvation in 12 members (*GmPHR7*, *9*, *12*, *14*, *19–22*, *24*, *25*, *32*, *33*), particularly for *GmPHR32*, for which transcription increased 5-fold in responses to P deficiency (Fig. 3B).

### Effects of P deficiency on transcription of *GmPHR* members in soybean nodules

**Fig. 4.**
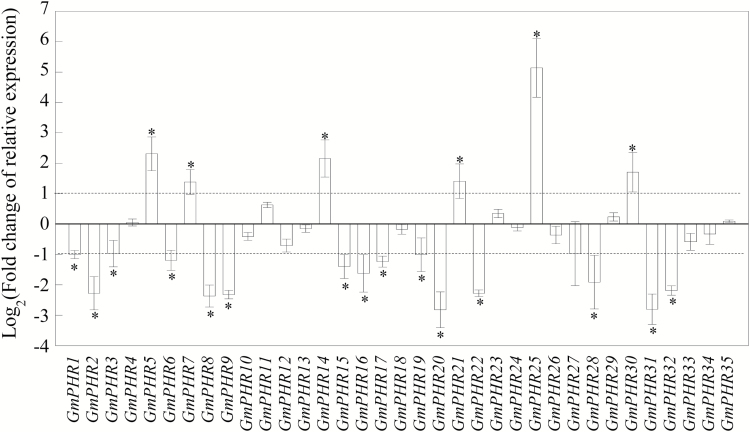
Expression patterns of *GmPHR* members in root nodules, expressed as the binary logarithm of fold-changes of relative expression of *GmPHR* members under high-P and low-P treatments. Data are mean of four replicates ±SE. *, Significant difference between high-P and low-P treatments (Student’s *t*-test, *P*<0.05).

Since soybean can form a special organ, the nodule, with rhizobium symbionts, the effects of P deficiency on the expression of *GmPHR* members in nodules were investigated by qPCR analysis. The results showed that transcripts of 21 members were significantly up-regulated or down-regulated by more than 2-fold in nodules under Pi deficiency conditions relative to those under P sufficient conditions (Fig. 4). Among them, expression levels were up-regulated for *GmPHR5*, *GmPHR7*, *GmPHR14*, *GmPHR21*, *GmPHR25*, and *GmPHR30*, especially for *GmPHR25* where transcripts were 30-fold higher in nodules under P deficiency. On the other hand, expression levels of 15 members (*GmPHR1–3*, *6*, *8*, *9*, *15–17*, *19*, *20*, *22*, *28*, *31*, *32*) decreased dramatically in response to P deficiency, with *GmPHR20* and *GmPHR31* transcripts decreased by 6-fold (Fig. 4). Expression levels of the other *GmPHR* members in nodules were relatively unaltered by P deficiency (Fig. 4). Using the housekeeping gene *GmACTIN* as a reference, similar expression patterns of eight *GmPHR* members in nodules were observed (Supplementary Fig. S6).

### Subcellular localization of GmPHR25

**Fig. 5.**
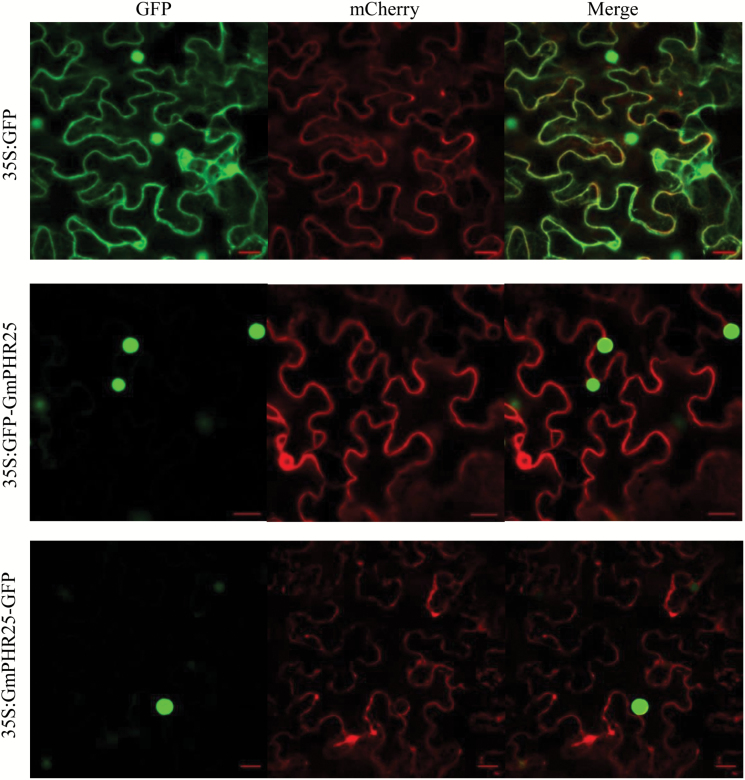
Subcellular localization of GmPHR25 fused to GFP protein in tobacco mesophyll cells: *35S*:*GFP*, *35S:GFP-GmPHR25*, *35S:GmPHR25-GFP*, and *35S:AtPIP2A*-*mCherry* fusion vectors are shown. GFP fluorescence and mCherry fluorescence were observed using confocal microscopy. Scale bars are 20 µm.

Since transcription of *GmPHR25* exhibited the most responses to P deficiency in all the soybean organs tested (i.e. leaves, roots, and nodules), and its MYB and Coiled-Coil domains were closely located on the N-terminus, which was different from most PHR members with well-known functions in plants (Fig. 1), GmPHR25 was selected for further analysis. To investigate the subcellular localization of GmPHR25, its encoding region was fused to GFP at either its N-terminus (GFP-GmPHR25) or C-terminus (GmPHR25-GFP), and the constructs were transiently expressed in tobacco leaves. Subcellular localization was examined by the detection of GFP signals (Fig. 5). Signals of the empty vector control were observed in the plasma membrane, cytoplasm, and nucleus (Fig. 5), whereas signals from fusion with GmPHR25 were only detected in the nucleus (Fig. 5), strongly suggesting that this is where GmPHR25 predominantly localizes.

### Overexpressing *GmPHR25* increases Pi concentration in soybean hairy roots *in vitro*

**Fig. 6.**
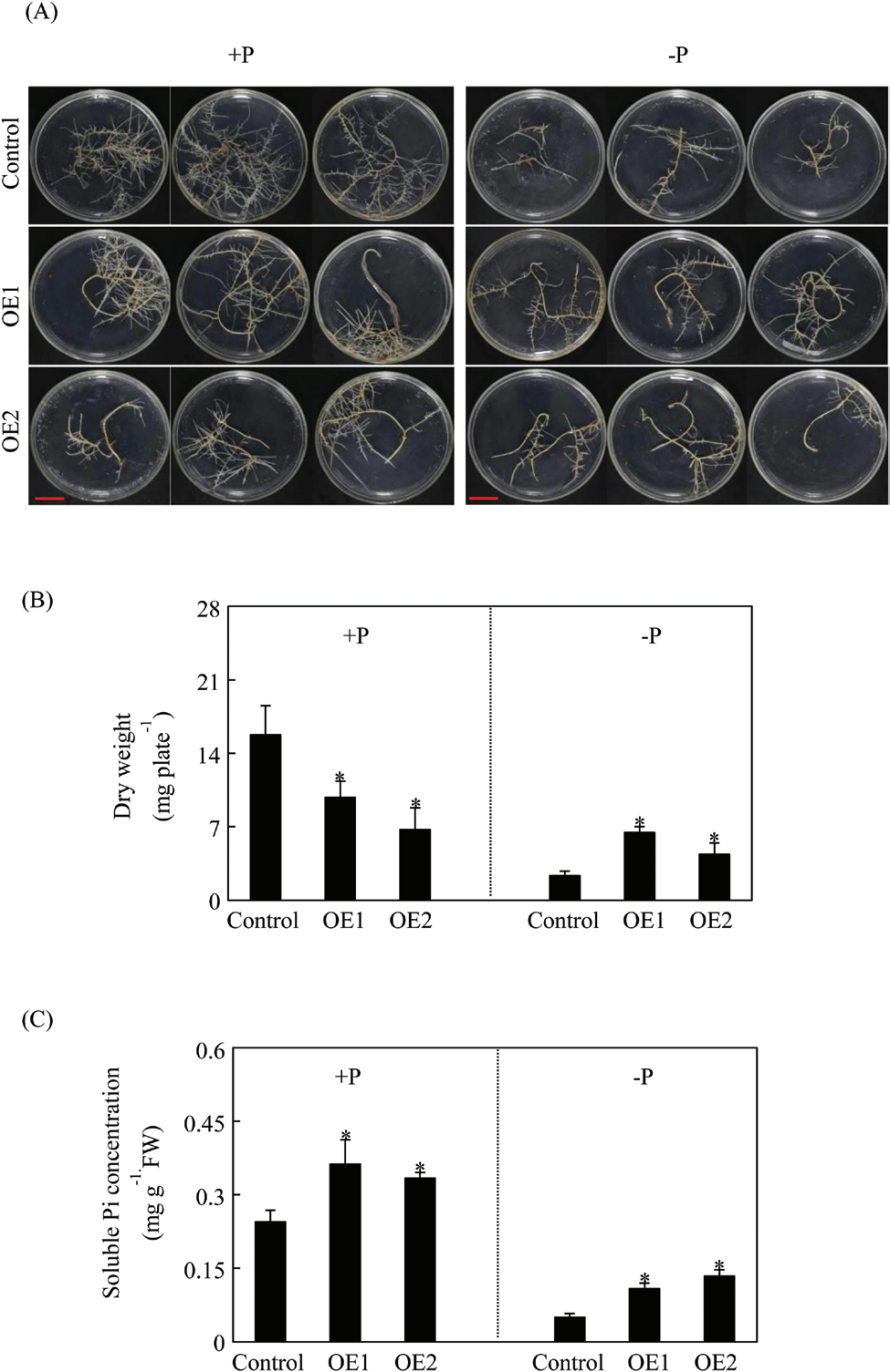
Dry weight and soluble Pi concentration in soybean hairy roots of control and *GmPHR25*-overexpressing plants. (A) Phenotype of hairy roots, (B) dry weight, and (C) soluble Pi concentration. Hairy roots were grown in MS medium for 14 d prior to transferring into MS medium containing 1.25 mM (+P) or 0 mM (–P) phosphorus. After a further 14 d, roots were harvested for analysis. Control represents hairy roots transformed with the empty vector; OE indicates transgenic hairy roots overexpressing *GmPHR25*. FW, fresh weight. Data are means of three replicates +SE. *, Significant difference between OE and control (Student’s *t*-test, *P*<0.05). Scale bars in (a) are 1 cm.

In order to assess *GmPHR25* functions in the control of plant Pi homeostasis, soybean hairy roots overexpressing *GmPHR25 in vitro* were generated. Increased expression of *GmPHR25* was verified through qPCR analysis, with *GmPHR25* expression levels being increased by more than 3-fold over empty vector control hairy roots (Supplementary Fig. S1A). Furthermore, *GmPHR25* overexpression significantly affected soybean hairy root growth and Pi concentration (Fig. 6). Overexpression of *GmPHR25* inhibited hairy root growth under P-sufficient conditions, as reflected by 37% and 57% decreases in root dry weights of the *GmPHR25* overexpression lines relative to the controls (Fig. 6B). However, overexpression of *GmPHR25* enhanced hairy root growth under P-deficient conditions, as reflected by 170% and 80% increases in hairy root dry weights (Fig. 6B). In contrast to changes in dry weight, relative to the control line, soluble Pi concertation in *GmPHR25* overexpression lines was increased by more than 30% (OE2 compared to control) and 110% (OE1 compared to control) under Pi-sufficient and -deficient conditions, respectively (Fig. 6C). Taken together, these results suggest that *GmPHR25* regulates soybean hairy root growth and Pi homeostasis.

### Functional analysis of *GmPHR25* in soybean composite plants

**Fig. 7.**
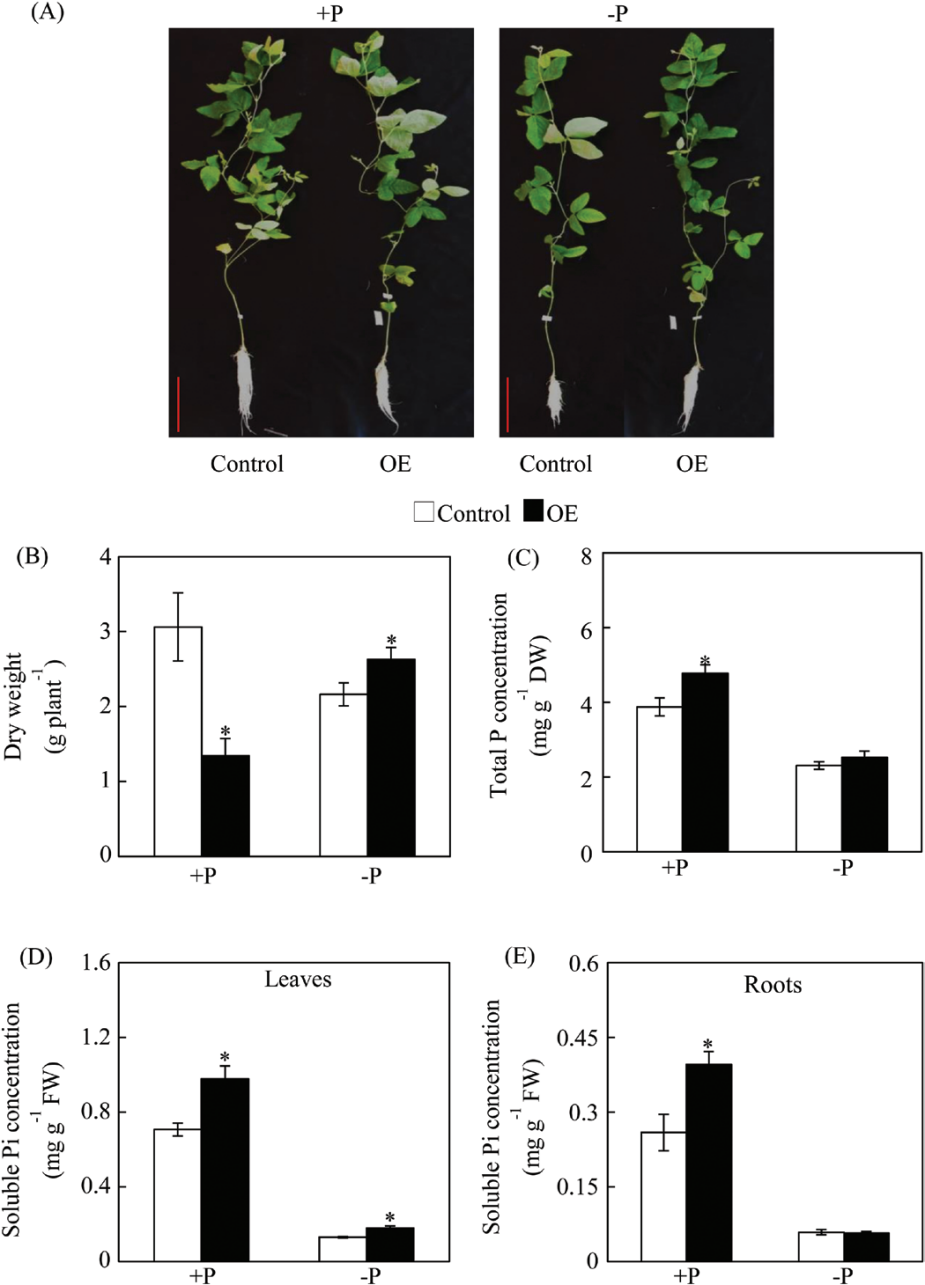
Dry weight and P concentration of control and *GmPHR25*-overexpressing composite soybean plants. (A) Phenotype of composite soybean plants, (B) dry weight, (C) total P concentration in the whole plant, (D) soluble Pi concentration of leaves, and (E) soluble Pi concentration of roots. Composite soybean plants with transgenic hairy roots were grown in normal nutrient solution for 14 d, then plants were transferred to nutrient solution containing 500 μM (+P) or 25 μM (–P) KH_2_PO_4_. After a further 14 d, shoots and roots were separately harvested for analysis. Control represents hairy roots transformed with the empty vector; OE indicates transgenic hairy roots overexpressing *GmPHR25*. DW, dry weight; FW, fresh weight. Data are means of six replicates ±SE. *, Significant differences between OE and control (Student’s *t*-test, *P*<0.05). Scale bars in (A) are 10 cm.

Functions of *GmPHR25* were further investigated in overexpressing soybean transgenic composite plants. Increased expression of *GmPHR25* in transgenic hairy roots was verified through qPCR analysis (Supplementary Fig. S1B). Under P-sufficient conditions, compared to control lines, overexpression of *GmPHR25* in soybean composite plants also resulted in a 56% decrease in plant dry weight (Fig. 7B), while total P concentration increased by 23% (Fig. 7C). More precisely, the soluble Pi concentration rose by 38% in leaves and by 52% in roots under Pi-sufficient conditions (Fig. 7D, E). However, under Pi-deficient condition, overexpression of *GmPHR25* only resulted in increased dry weight and soluble Pi concentration in leaves, compared with the control lines (Fig. 7B, D). These results further reinforce the suggestion that *GmPHR25* affects Pi homeostasis in plants. However, it was observed that suppressed *GmPHR25* expression did not affect dry weight and P concentration for transgenic composite plants, except for decreased soluble Pi concentrations in leaves under P-sufficient conditions (Supplementary Fig. S2), suggesting that function redundancy might be present for GmPHR25 in soybean.

**Fig. 8.**
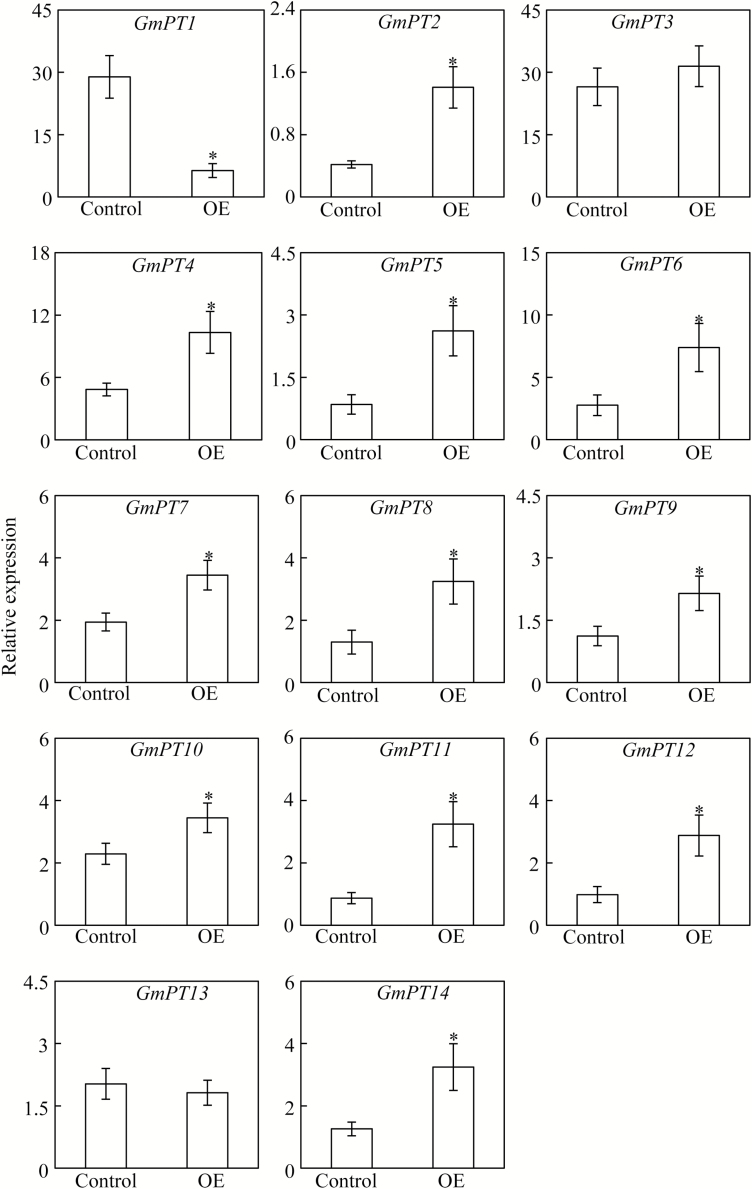
Transcripts of *GmPT*s in *GmPHR25*-overexpressing composite soybean plants. Plants were grown in nutrient solution containing 500 μM KH_2_PO_4_ for 14 d, and transcripts in hairy roots were determined by qPCR. Control represents soybean hairy roots transformed with the empty vector; OE indicates transgenic soybean hairy roots overexpressing *GmPHR25*. Data are means of six replicates ±SE. *, Significant differences in downstream gene expression between OE and control plants (Student’s *t*-test, *P*<0.05).

**Fig. 9.**
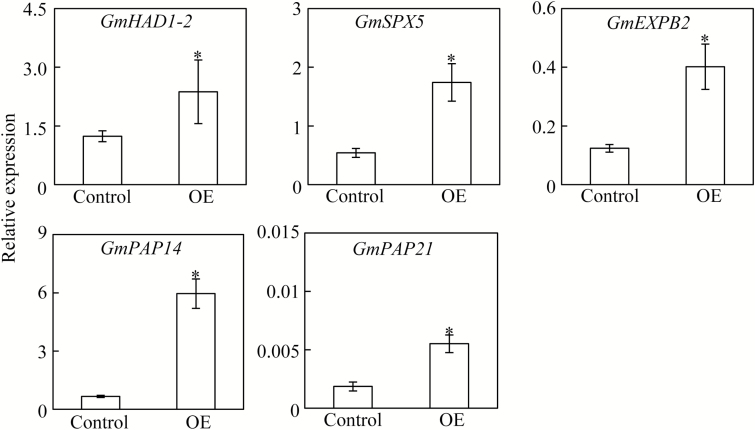
Expression of five Pi starvation-responsive genes in *GmPHR25*-overexpressing composite soybean plants. Plants were grown in nutrient solution containing 500 μM KH_2_PO_4_ for 14 d, and transcripts of candidate genes in hairy roots were determined by qPCR. Control represents soybean hairy roots transformed with the empty vector; OE indicates transgenic soybean hairy roots overexpressing *GmPHR25*. Data are means of six replicates ±SE. *, Significant differences in gene expression between OE and control plants (Student’s *t*-test, *P*<0.05).

To further elucidate the regulatory roles of *GmPHR25* in soybean, transcription of 14 high-affinity Pi transporters (*GmPT*), five Pi starvation-responsive genes, and 34 other *GmPHR* members were analysed in hairy roots of transgenic composite plants. Except for *GmPT1*, *GmPT3*, and *GmPT13*, transcripts of other *GmPT* members were significantly increased in composite plant hairy roots overexpressing *GmPHR25*, with expression of *GmPT2* increased over 2-fold (Fig. 8). Consistently, at least one P1BS element could be detected in the promoter regions of *GmPT* members, including *GmPT2*, *GmPT5*, *GmPT8*, *GmPT9*, *GmPT10*, and *GmPT12* (Supplementary Table S4). These results indicate that *GmPHR25* regulates *GmPT* expression patterns, and thus controls Pi homeostasis in soybean. In addition, overexpression of *GmPHR25* significantly increased the expression levels of five Pi starvation-responsive genes, namely *GmHAD1-2*, *GmSPX5*, *GmEXPB2*, *GmPAP14*, and *GmPAP21* (Fig. 9). However, except for significant increases of *GmPHR8* and *GmPHR22* expression levels (Supplementary Fig. S7), overexpression of *GmPHR25* had no effect on the transcription levels of the other 32 *GmPHR* members (data not shown). These results strongly suggest that *GmPHR25* plays an important role in the P signaling network in soybean.

## Discussion

The critical roles of *PHR1* and *PHR1-like* genes in the P signaling network have been well elucidated in several plant species, including Arabidopsis, rice, bean, wheat, maize, and rape (Rubio *et al.*, 2001; Valdés-López *et al.*, 2008; Zhou *et al.*, 2008; Ren *et al.*, 2012; Wang *et al.*, 2013*a*, 2013*b*; Sun *et al.*, 2016; Ruan *et al.*, 2017). Furthermore, all PHR1 members with known functions have been characterized as having the MYB and Coiled-Coil domains localization at their C-terminus, except for PvPHR1 in bean, AtPHL2 and AtPHL3 in Arabidopsis (Fig. 1). However, expression patterns of *PHR* family members in soybean and functions of GmPHR members exhibiting the MYB and Coiled-Coil domains at the N-terminus remain unclear. With the release of soybean genome sequences, it is now possible to characterize *GmPHR* members and dissect their involvement in adaptations to P deficiency. In the current study, gene structures and expression patterns of *GmPHR* members were analysed in soybean for the first time. Furthermore, the roles of *GmPHR25* in controlling Pi homeostasis were investigated through functional analysis in transgenic hairy roots *in vitro* and *in vivo*.

In total, 35 *GmPHR* members were identified in the soybean genome via BLAST searches on the phytozome website. All GmPHR members could be further divided into two groups through phylogenetic analysis (Fig. 1). It was found that 11 soybean GmPHR members belong to group II, which also contains representatives from other species known to function as key regulators in P signaling pathways, including AtPHR1 and AtPHL1 in Arabidopsis, OsPHR2 in rice, BnPHR1 in rape, ZmPHR1 in maize, and TaPHR1 in wheat (Rubio *et al.*, 2001; Valdés-López *et al.*, 2008; Zhou *et al.*, 2008; Ren *et al.*, 2012; Wang *et al.*, 2013*a*, 2013*b*; Guo *et al.*, 2015). The other 24 GmPHR members were more homologous to PvPHR1 in common bean (Fig. 1). Among two the GmPHR groups, each PHR member contains at least one copy, and presents as duplicated pairs, such as GmPHR1 and GmPHR16, GmPHR7 and GmPHR30, GmPHR6 and GmPHR15, GmPHR8 and GmHR31 (Fig. 1). Consistent with this, it has been suggested that soybean has experienced at least two rounds of whole-genome duplication, thus resulting in approximately 75% of its genes being present in multiple copies (Shoemaker *et al.*, 2006; Schmutz *et al.*, 2010). Furthermore, diverse functions of duplicated genes in soybean have been suggested, such as *GmCHLI* controlling chlorophyll biosynthesis and *GmTfl1* controlling growth habit (Tian *et al.*, 2010; Li *et al.*, 2016). It was observed that several *GmPHR* duplicated pairs exhibit different expression patterns, suggesting diverse functions present in GmPHR paralogs in soybean. For example, *GmPHR6* exhibited high expression levels in both roots and pods (Fig. 2); however, its duplicated paralog *GmPHR15* only exhibited relatively high transcripts in roots. *GmPHR7* expression was significantly increased by P deficiency in roots, but this was not the case for its duplicated paralog *GmPHR30* (Fig. 3B).

Genome-wide transcriptomic analysis in soybean has revealed transcripts of *GmPHR* members in various tissues, including leaves, roots, nodules, flowers, pods, and seeds, which are summarized in Supplementary Table S1 (Libault *et al.*, 2010; Severin *et al.*, 2010). Consistent with these published results, transcripts of *GmPHR* members were also detected in the tissues tested in the current study through qPCR analysis using their specifc primers, including leaves, roots, flowers, pods, and seeds (Fig. 2, Supplementary Fig. S8). However, relative expression levels of several *GmPHR* members differed from previous transcriptomics results. For example, the expression of *GmPHR10* and *GmPHR14* in the current study was highest in leaves (Fig. 2), whereas previous studies indicate it was highest in roots (Supplementary Table S1). These inconsistencies might be the result of differences in experimental materials, growth conditions, or analytical techniques. Despite these differences, the observation that *GmPHR* members are widely expressed throughout soybean, with variations among tissues, remains valid and is largely consistent with published reports.

In this study, P deficiency significantly increased expression levels of most *GmPHR* members (Fig. 3B), and demonstrated that responses vary among members. Similar results have also been observed in other plant species. For example, in Arabidopsis, expression levels of two *PHR* members (*AtPHL2* and *AtPHL3*) were significantly increased by Pi starvation, while two other members (*AtPHR1* and *AtPHL1*) exhibited no responses (Rubio *et al.*, 2001; Sun *et al.*, 2016). In rice, P deficiency resulted in significantly increased transcription of *OsPHR3* and *OsPHR4*, but not *OsPHR1* or *OsPHR2* (Zhou *et al.*, 2008; Guo *et al.*, 2015; Ruan *et al.*, 2017). These results strongly suggest that regulatory mechanisms underlying *PHR* member responses to P deficiency vary among these members, which warrants further study of the underlying mechanisms.

Further analysis of expression patterns of *GmPHR* members in both leaves and roots under N deficiency conditions revealed that several Pi starvation-responsive *GmPHR* members also respond to N deficiency in both leaves and roots (Fig. 3A). For example, in roots, expression of Pi starvation-responsive *GmPHR14* and *GmPHR19* increased by 2.3 and 3.1-fold, respectively, under N deficiency conditions (Fig. 3). These results indicate potential crosstalk in plant responses to P and N deficiencies. Recently, global transcriptome profiling has demonstrated that a large number of genes are responsive to both N and P deficiencies (Cai *et al.*, 2013; Takehisa *et al.*, 2015). For example, it was found that 159 genes in roots and 101 genes in shoots were up-regulated after 7 d of both N and P deficiencies, with one notable example being *MYB101* (a MYB transcription factor) in rice (Cai *et al.*, 2013). Furthermore, several key regulators have been suggested to play crucial roles in both N and P signaling in plants. For example, AtNLA is considered a key regulator of Arabidopsis adaptations to N-limiting conditions, because the *nla* mutant fails to develop essential adaptive responses to N limitation, and thus exhibits early and rapid senescence (Peng *et al.*, 2007). Meanwhile, AtNLA can recruit PHOSPHAT2 (PHO2) to degrade PT2, and thus control Pi homeostasis in Arabidopsis (Kant *et al.*, 2011; Park *et al.*, 2014). Therefore, it seems that soybean responses to P and N deficiencies share common signaling pathway elements, which might be regulated by GmPHR members.

Soybean can interact with rhizobia, and thus form symbiotic associations, which significantly affects N and P acquisition and utilization by the plant. Recently, it has been shown that rhizobium inoculation not only improves N nutrition, but also influences P acquisition and utilization in soybean (Cheng *et al.*, 2009; Qin *et al.*, 2011; Ding *et al.*, 2012). Rhizobium inoculation resulted in significant increases in exudation of protons and organic acids, and thus enhanced the capability of soybean to mobilize Ca-P and Al-P (Qin *et al.*, 2011; Ding *et al.*, 2012). Furthermore, Pi starvation-responsive *GmPT5* and *GmEXPB2* have been documented to control soybean Pi homeostasis and nodule development, respectively (Qin *et al.*, 2012*b*; Li *et al.*, 2015). It is therefore suggested that nodules contain adaptive strategies for responding to Pi starvation. Consistent with this hypothesis, expression levels of six *GmPHR* members (*GmPHR5*, *GmPHR7*, *GmPHR14*, *GmPHR21*, *GmPHR25*, and *GmPHR30*) were significantly increased by P deficiency in nodules, with *GmPHR25* being particularly notable (Fig. 4). Furthermore, expression levels of *GmEXPB2* and *GmPT5* were up-regulated by *GmPHR25* overexpression in soybean hairy roots (Figs 8, 9), which strongly suggests that *GmPHR25* is involved in regulating Pi homeostasis in the nodules.

The role of AtPHR1 and its orthologues in P signaling and homeostasis has been well established in several plant species, which indicates that members of this gene family share a conserved function as central regulators in plant Pi homeostasis (Rubio *et al.*, 2001; Valdés-López *et al.*, 2008; Zhou *et al.*, 2008; Bustos *et al.*, 2010; Ren *et al.*, 2012; Wang *et al.*, 2013*a*, 2013*b*). In this study, *GmPHR25* was selected as a candidate for further functional analysis because it exhibits the highest sequence similarity to *PvPHR1*, higher Pi starvation responses in leaves, roots, and nodules than other *GmPHR* members, and it is localized to the nucleus (Fig. 5). It was observed that *GmPHR25* overexpression increased soluble Pi concentration in transgenic soybean hairy roots *in vivo* and *in vitro* under Pi-sufficient conditions (Figs 6, 7), which strongly supports the hypothesis that *GmPHR25* acts in controlling Pi homeostasis. Furthermore, accompanied by increased Pi concentration, decreased growth in transgenic lines with *GmPHR25* overexpression was observed under Pi-sufficient conditions (Figs 6, 7), strongly suggesting excessive Pi accumulation might inhibit plant growth. Consistent with this, similar results have also been found in rice through overexpressing *OsPHR2* and *OsPHR4* (Zhou *et al.*, 2008; Ruan *et al.*, 2017). However, the molecular mechanisms underlying a significant decrease in plant growth with excessive Pi accumulation remain unknown.

Since Pi transporters play important roles in Pi uptake and translocation in plants, expression patterns of soybean Pi transporters (*GmPT*) were assayed by qPCR analysis in hairy roots overexpressing *GmPHR25*. As a result of *GmPHR25* overexpression, transcription increased for most *GmPT* members, except *GmPT1*, *GmPT3*, and *GmPT13* (Fig. 8), which strongly suggests that GmPHR25 regulates *GmPT* expression, and thus controls Pi homeostasis (Figs 6, 7). Within plant signaling networks, it has been well documented that several PT members act downstream of PHR1 (Chiou and Lin, 2011; Liang *et al.*, 2014). For example, transcript levels of *AtPht1;4*, *AtPht1;7*, *AtPht1;8*, and *AtPht1;9* were all significantly increased by *AtPHR1* overexpression in Arabidopsis (Nilsson *et al.*, 2007). In rice, *OsPT1*, *OsPT5*, *OsPT7*, *OsPT9*, and *OsPT12* have also been placed downstream of *OsPHR2*, because overexpression or suppression *OsPHR2* leads to respective increases or decreases in their expression levels (Zhou *et al.*, 2008). Although the functions of most *GmPT* members remain largely unknown, with the exception of *GmPT5*, it is reasonable to conclude that their expression levels influence Pi homeostasis in soybean. Beyond Pi transporters, transcripts of other Pi starvation-responsive genes were also increased by *GmPHR25* overexpression in soybean hairy roots. The Pi-responsive genes tested here included *GmEXPB2*, *GmSPX5*, *GmHAD1-2*, *GmPAP14*, and *GmPAP21* (Fig. 9). Among them, *GmEXPB2* has been suggested to play a critical role in soybean root and nodule growth, possibly through cell wall modification, thereby affecting Pi acquisition and accumulation (Guo *et al.*, 2011; Li *et al.*, 2015). Recently, *GmPAP21* has been suggested to be involved in P utilization in soybean nodules (Li *et al.*, 2017). Therefore, it is possible that GmPHR25 is a critical regulator in controlling Pi acquisition and uptake in soybean through effects on the transcription of Pi starvation-responsive genes. However, unlike AtPHR1 or OsPHR2, GmPHR25 did not harbor any transcriptional activity in yeast (Supplementary Fig. S3). Since OsPHR2 transcription activity is determined by 230 amino acids at its N-terminus in the yeast analysis system (Zhou *et al.*, 2008), which are lacking in GmPHR25 (Fig. 1), it might be plausible that GmPHR25 exhibits no transcription activity, and thus is different from OsPHR2. Furthermore, GmPHR25 exhibited binding activity against the synthetic fragment containing four P1BS elements despite having no binding activity against the promoters of *GmPT9*, *GmPT10*, and *GmPT12* (Supplementary Figs S4, S5). These results strongly suggest that GmPHR25 might regulate transcription of its downstream genes through interaction with other regulators. Consistent with this, it has recently been reported that OsPHR4 could interact with other OsPHR members to regulate transcription of downstream genes in rice (Ruan *et al.*, 2017).

In summary, *GmPHR* members were systematically characterized, including expression patterns among tissues, and responses to nutrient deficiencies (i.e. N and P). In addition, evidence placing *GmPHR25* as a regulator in the P signaling network has also been presented. These data provide not only a comprehensive list of *GmPHR* members in soybean, but also information on their properties, as well as results confirming their roles in the soybean P signaling network.

## Supplementary data

Supplementary data are available at *JXB* online.

Fig. S1. Expression of *GmPHR25* in soybean hairy roots.

Fig. S2. Dry weight, total P concentration and soluble Pi concentration of control and RNAi*-GmPHR25* in composite soybean plants.

Fig. S3. Transcriptional activity analysis of GmPHR25 in yeast.

Fig. S4. Yeast one-hybrid analysis of the DNA-binding affinity of GmPHR25 for the *4xP1BS* module.

Fig. S5. Yeast one-hybrid analysis of the DNA-binding affinity of GmPHR25 for the *GmPT9*, *GmPT10*, and *GmPT12* promoters.

Fig. S6. Expression patterns of eight *GmPHR* members and *GmEF1-α* in nodules at two P levels.

Fig. S7. Transcripts of *GmPHR8* and *GmPHR22* in overexpressing *GmPHR25* composite soybean plants.

Fig. S8. Amplification efficiency for each pair of of *GmPHR*-specific primers for qRT-PCR analysis.

Table S1. The expression profiles of *GmPHR* members from the SoyBase (http://soybase.org/soyseq/).

Table S2. Primer sequences used in this study for qPCR.

Table S3. Primer sequences used in this study for vector construction.

Table S4. Analysis of the putative PHR1-binding site in a 3.0-kb sequence upstream of the start codon of each *GmPT* member.

## Supplementary Material

Supplementary FiguresS1-S8 and Tables S1-S4
