# Supplementary material for: GmPHR25, a GmPHR member up-regulated by phosphate starvation, controls phosphate homeostasis in soybean
Source: J Exp Bot. 2017 Aug 23;68(17):4951–67. doi: 10.1093/jxb/erx292 (PMC5853305; doi:10.1093/jxb/erx292)
Supplement: Supplementary FiguresS1-S8 and Tables S1-S4 [file erx292_suppl_supplementary_figures_s1_s8_tables_s1_s4.pdf]

Fig. S1.

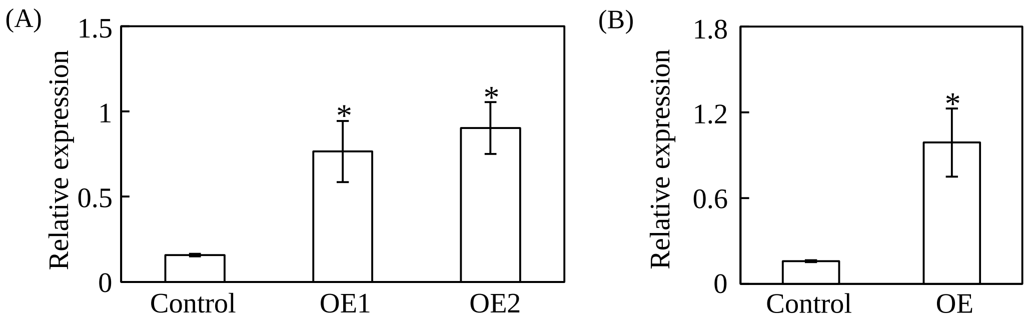

Fig. S1. Expression of *GmPHR25* in soybean hairy roots. (A) Transcripts of *GmPHR25* in soybean hairy roots *in vitro*. (B) Expression levels of *GmPHR25* in hairy roots of soybean composite plants. Control represents soybean hairy roots transformed with the empty vector; OE means transgenic soybean hairy roots with overexpressing *GmPHR25*. Each bar is the mean of three (A) or six (B) replicates with the standard error. Asterisk indicated significant difference between OE and control through Student's *t*-test (*: $P<0.05$).

Fig. S2.

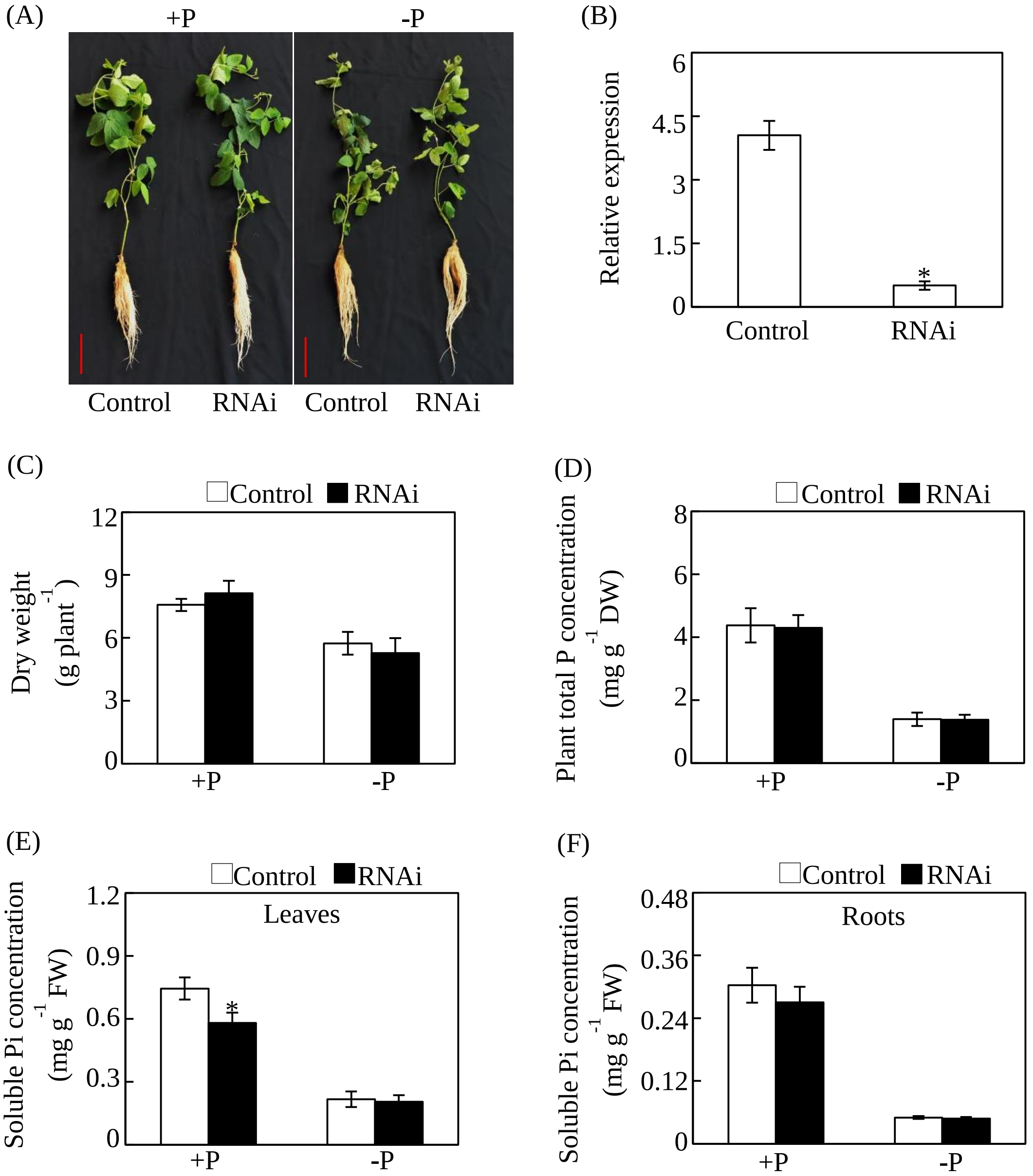

Fig. S2. Dry weight, total P concentration and soluble Pi concentration of control and RNAi-*GmPHR25* in composite soybean plants. (A) Phenotype of composite soybean plants. (B) Expression of *GmPHR25* in soybean hairy roots. (C) Dry weight. (D) Plant total P concentration. (E) Soluble Pi concentration of leaves. (F) Soluble Pi concentration of roots. Composite soybean plants with transgenic hairy roots were grown in normal nutrient solution for 14 d. Then, plants were transferred into nutrient solution containing 500 µM (+P) or 25 µM (-P) KH_2PO_4. After 14 d, shoots and roots were separately harvested for analysis. Control represents soybean hairy roots transformed with the empty vector; OE indicates transgenic soybean hairy roots overexpressing *GmPHR25*. DW and FW represent dry weight and fresh weight, respectively. Each bar is mean of six replicates with the standard error. Asterisks indicate significant differences between RNAi and control in Student's *t*-test (*: $P<0.05$). Bar = 10 cm.

Fig. S3.

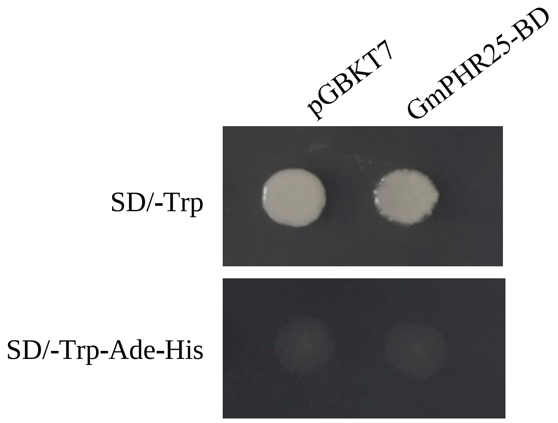

Fig. S3. Transcriptional activity analysis of GmPHR25 in yeast. Full-length *GmPHR25* was cloned into the *pGBKT7* (*BD*) vector and transformed into yeast strain AH109. *pGBKT7* empty vector was used as negative control. The minimal medium SD/-Trp or SD/-Trp -Ade-His was used for selection.

Fig. S4

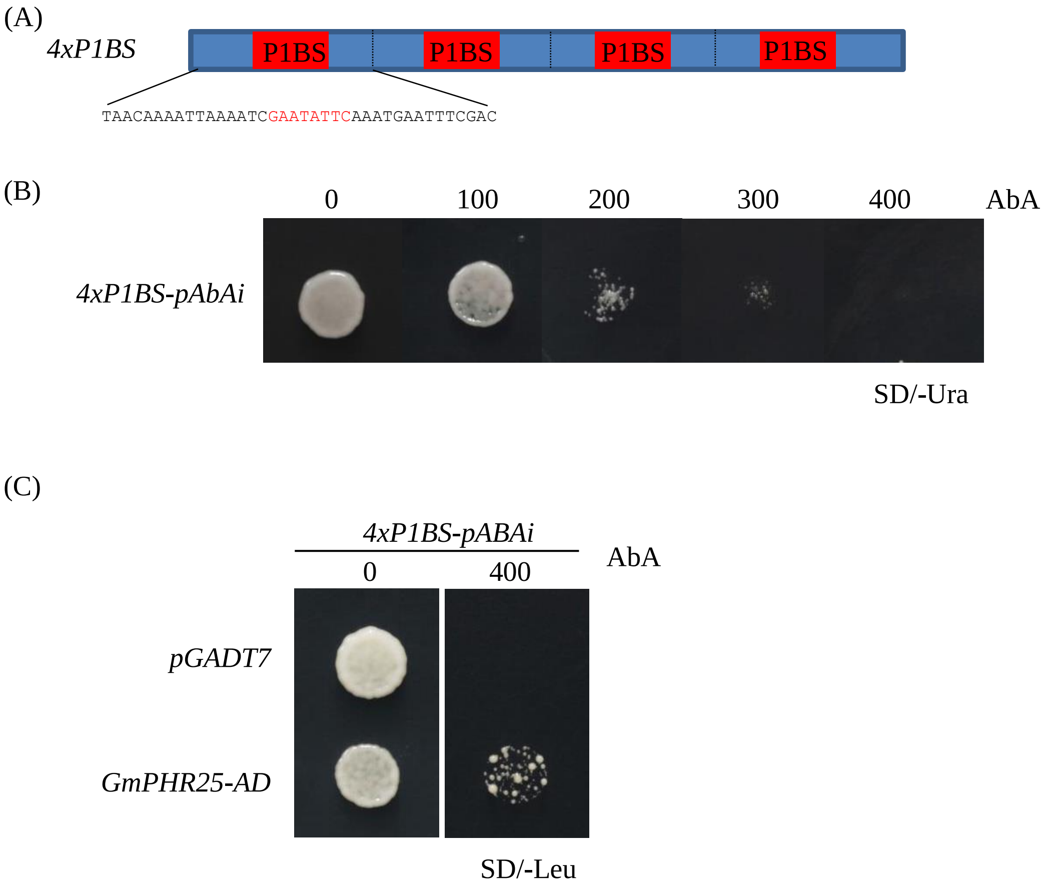

Fig. S4. Yeast one-hybrid analysis of the DNA binding affinity of GmPHR25 for *4xP1BS* module. (A) The schematic diagram of *4xP1BS* module. The red box indicated the P1BS motif and was quadruplicated in tandem. The *4xP1BS* module was cloned into *pABAi* vector, then transformed and integrated into yeast Y1HGold genome. (B) The minimal inhibitory concentration (ng/µL) of AbA (Aureobasidin A) for bait strain of *4xP1BS-pAbAi*. (C) The interaction analysis between GmPHR25 and *4xP1BS* module. Full-length GmPHR25 was cloned into the *pGADT7* (*AD*) vector and transformed into yeast bait strain of *4xP1BS-pAbAi*. *pGADT7* empty vector was used as negative control. The minimal medium SD/-Ura or SD/-Leu was used for selection.

Fig.S5

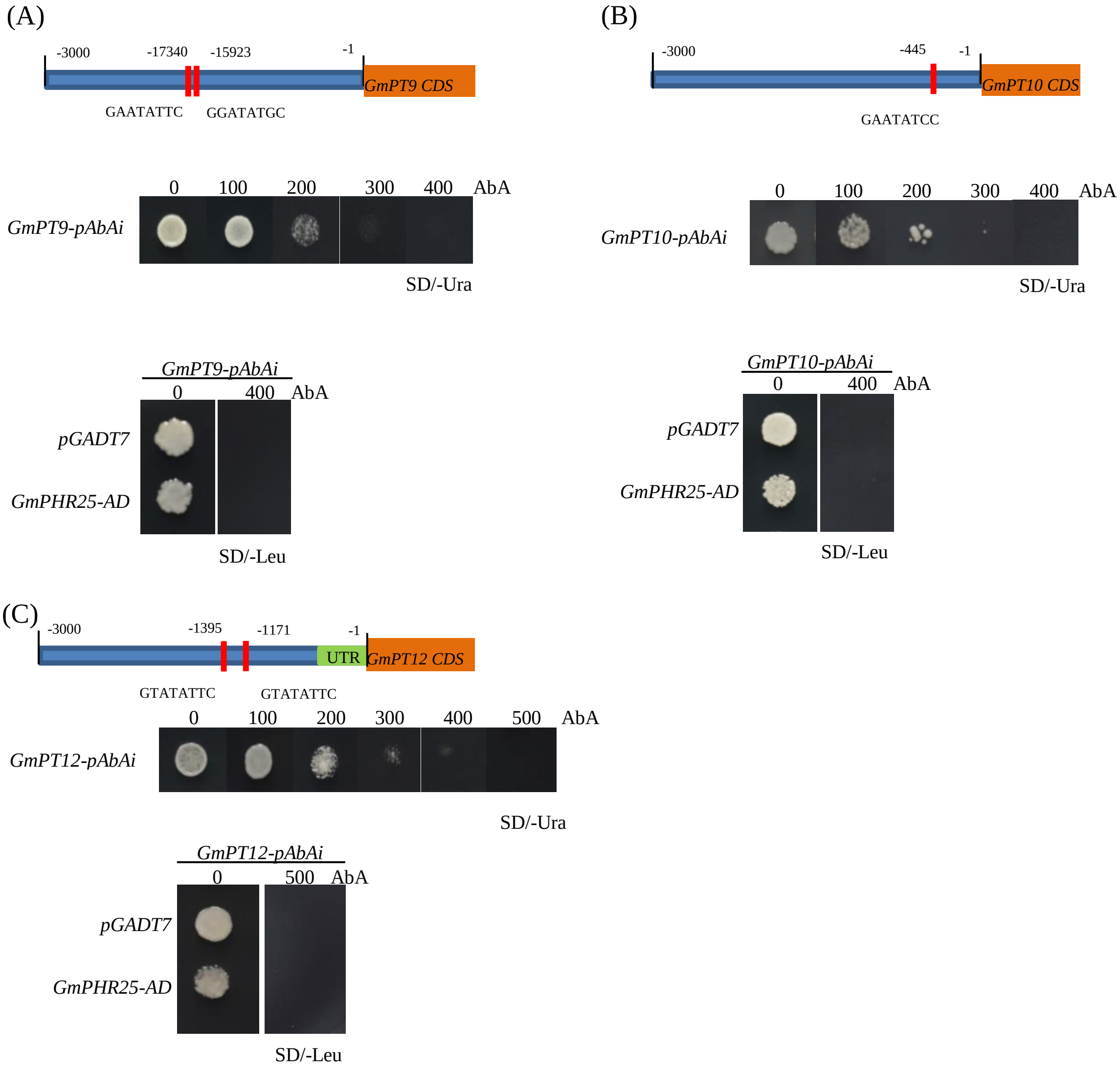

Fig. S5. Yeast one-hybrid analysis of the DNA binding affinity of GmPHR25 for *GmPT9*, *GmPT10* and *GmPT12* promoters. Red box indicated the P1BS motifs in the schematic diagram of *GmPT9* (A), *GmPT10* (B) and *GmPT12* (C) promoters. The sequences about 400 bp contain the P1BS motifs was cloned into the *pABAi* vector then transformed and integrated into yeast Y1HGold genome, respectively. The Aureobasidin A (AbA) resistance was analyzed for bait strain of *GmPT9-pAbAi*, *GmPT10-pAbAi* and *GmPT12-pAbAi* on the SD/-Ura medium. Full-length *GmPHR25* was cloned into the *pGADT7* (*AD*) vector and transformed into the bait strains and selected on the SD/-Leu medium contenting minimal inhibitory concentration (ng/ μL) of AbA for the interaction assay. *pGADT7* empty vector was used as negative control.

Fig. S6

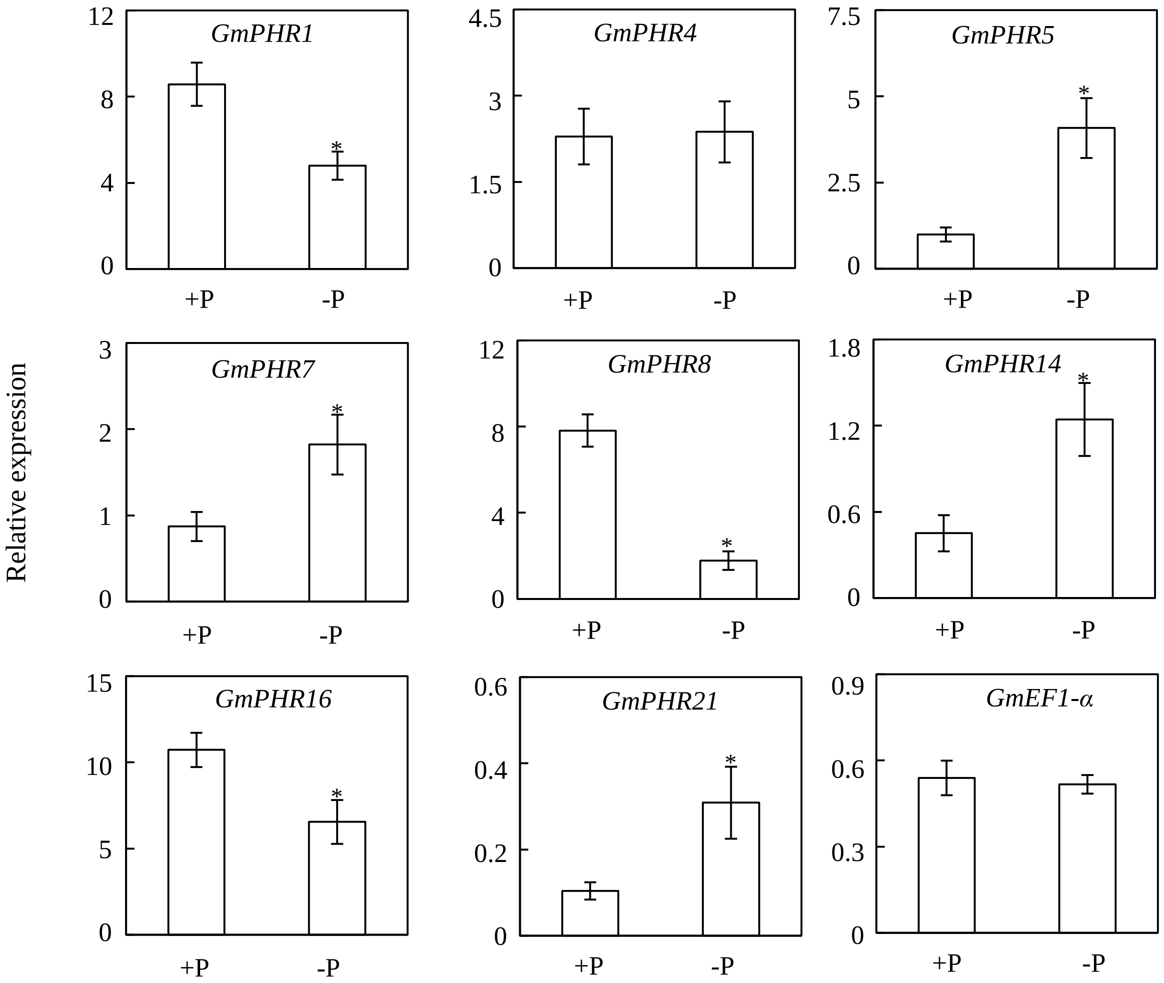

Fig. S6. Expression patterns of eight *GmPHR* members and *GmEF1-α* in nodules at two P levels. After 7 d of germination, soybean seedlings were inoculated with rhizobium, *Bradyrhizobium sp*. BXYD3 for 1 hour (h), and then transplanted into low nitrogen (100 μM total N) nutrient solution containing 5 μM (-P) or 500 μM (+P) KH_2PO_4. Nodules were harvested at 30 d after inoculation for qRT-PCR analysis. *GmACTIN* was used as housekeeping gene. Data in the figure are mean of four replicates with standard error bars. Asterisks indicate significant difference between +P and -P treatment in Student's *t*-test (*: $P<0.05$).

Fig. S7

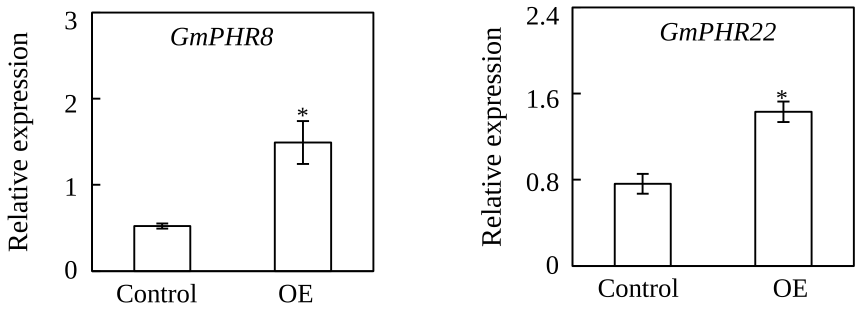

Fig. S7. Transcripts of *GmPHR8* and *GmPHR22* in overexpressing *GmPHR25* composite soybean plants. Soybean composite plants were grown in nutrient solution containing 500 µM KH_2PO_4 for 14 d. Transcripts of *GmPHR8* and *GmPHR22* in hairy roots were determined by qPCR. Control represents soybean hairy roots transformed with the empty vector; OE indicates transgenic soybean hairy roots overexpressing *GmPHR25*. Each bar is mean of six replicates with the standard error. Asterisks indicate significant differences between OE and control in Student's *t*-test (*: $P<0.05$).

Fig. S8

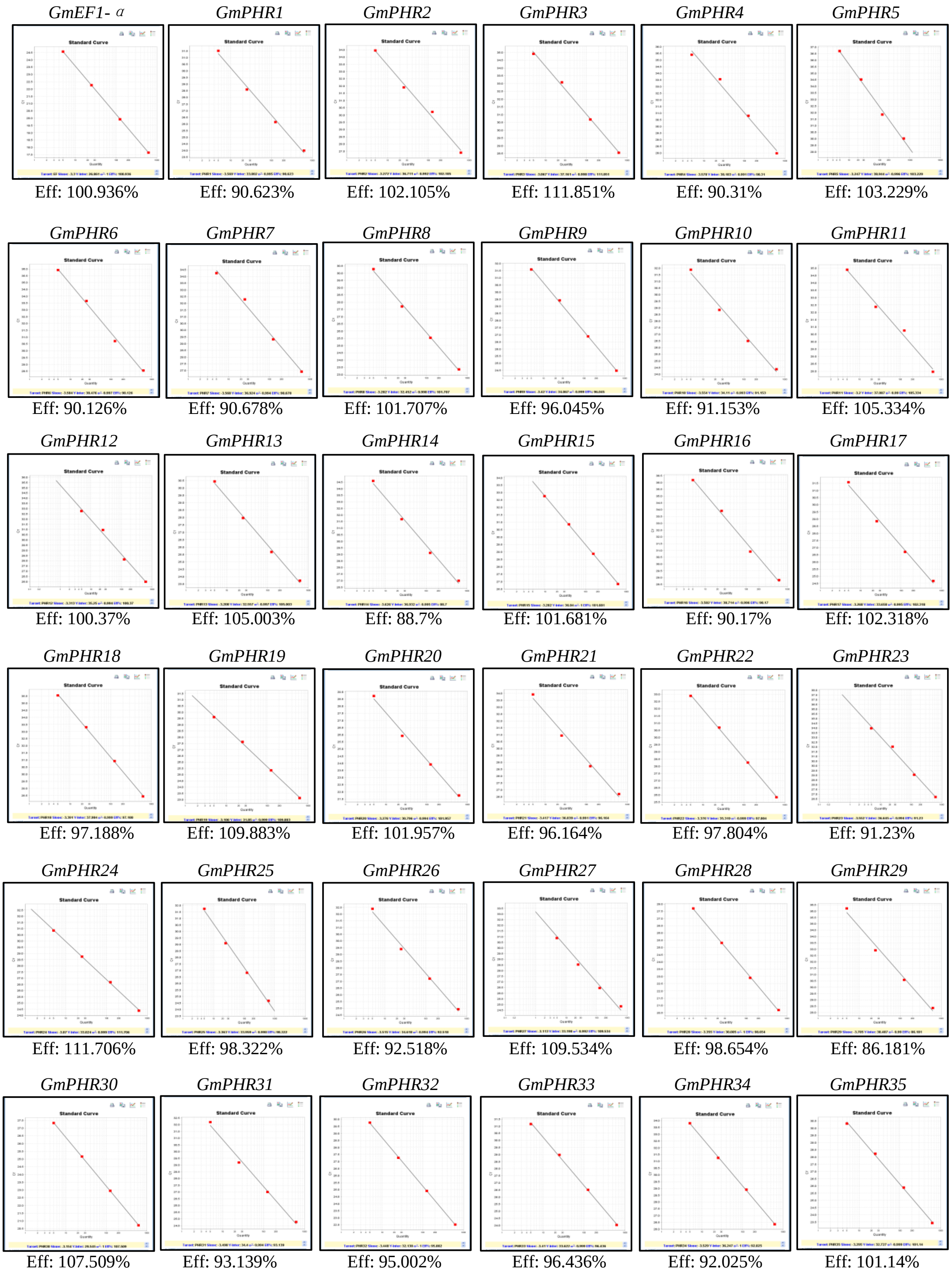

Fig. S8. Amplification efficiency for each pair of *GmPHR* specific primers for qRT-PCR analysis. Eff: efficiency.

Table S1. The expression profiles of *GmPHR* members from the SoyBase (http://soybase.org/soyseq/)

Gene	Locus	Young leaf	flower	one cm pod	pod shell 10DAF	pod shell 14DAF	seed 10DAF	seed 14DAF	seed 21DAF	seed 25DAF	seed 28DAF	seed 35DAF	seed 42DAF	root	nodule
GmPHR1	Glyma01g01300	5	7	8	7	5	25	9	3	4	1	7	2	29	170
GmPHR2	Glyma01g05920	25	18	25	20	10	13	10	6	10	12	40	16	55	44
GmPHR3	Glyma02g07790	1	1	1	1	0	0	0	0	0	0	3	3	11	0
GmPHR4	Glyma02g12070	38	18	10	12	5	3	4	1	2	5	11	7	13	39
GmPHR6	Glyma02g30800	0	3	1	1	4	3	3	1	3	7	5	3	27	6
GmPHR7	Glyma03g00590	45	44	25	35	20	18	11	4	12	4	13	3	52	53
GmPHR8	Glyma03g29940	0	11	0	1	3	1	4	1	1	2	0	2	82	199
GmPHR9	Glyma03g32350	84	111	65	55	49	32	22	13	40	18	32	14	67	238
GmPHR10	Glyma03g41040	28	45	20	37	16	28	35	5	36	12	18	16	84	74
GmPHR11	Glyma07g35700	5	21	6	7	1	12	4	5	9	6	18	6	12	1
GmPHR12	Glyma08g17400	9	29	12	14	8	3	0	1	2	0	2	5	26	0
GmPHR13	Glyma09g02030	94	72	103	134	101	53	79	31	97	84	134	114	201	204
GmPHR14	Glyma09g02040	166	140	129	145	90	60	72	43	92	60	51	27	204	105
GmPHR17	Glyma10g04540	4	4	3	1	1	6	3	0	14	8	27	17	41	12
GmPHR18	Glyma10g34050	344	71	106	134	84	47	112	56	152	74	104	49	81	51
GmPHR19	Glyma11g18990	2	34	5	7	6	1	0	1	0	0	1	1	32	0
GmPHR20	Glyma12g09490	2	101	11	13	4	1	1	2	2	4	11	1	14	1
GmPHR21	Glyma12g31020	5	47	1	6	1	0	0	0	0	0	1	0	13	0
GmPHR23	Glyma13g39290	5	16	0	3	1	0	0	0	0	0	0	3	2	0
GmPHR24	Glyma15g12930	154	105	161	165	133	72	86	45	106	77	121	80	281	91
GmPHR25	Glyma15g12940	139	130	126	140	87	99	75	31	75	62	59	22	253	88
GmPHR26	Glyma15g29620	5	27	7	10	8	3	0	0	0	0	2	1	25	3
GmPHR27	Glyma15g41740	8	32	17	16	8	1	0	2	0	0	6	2	21	2
GmPHR28	Glyma16g26820	0	12	1	2	5	0	0	1	0	0	0	0	19	0
GmPHR29	Glyma18g43130	0	2	0	1	2	1	0	0	0	1	1	0	4	9
GmPHR30	Glyma19g30220	51	18	28	50	37	48	18	5	28	12	16	12	37	52
GmPHR31	Glyma19g32850	0	36	2	2	4	1	0	0	0	0	0	0	67	148
GmPHR32	Glyma19g35080	65	49	50	42	31	10	23	10	30	18	52	17	80	296
GmPHR33	Glyma19g43690	39	41	19	33	10	14	16	3	11	18	31	11	53	67
GmPHR34	Glyma20g04630	13	14	22	36	9	8	7	2	4	9	15	10	23	4
GmPHR35	Glyma20g33540	244	104	70	101	48	38	113	47	103	36	79	23	31	27

Table S2. Primer sequences used in this study for qPCR

Gene	Forward primer (5'-3')	Reverse primer (5'-3')
GmPHR1	TTAGTAATCGTTCTTTGGCACC	TCTTCTGTACCTTTAGCTGCTC
GmPHR2	GAGGTGCAGATAAAGCAACTC	ATTTGCATTTGGAGTGCCTG
GmPHR3	ATGATGCAAGGAGGCAATGG	TGATCCTGTACTTCTGTAGATGAC
GmPHR4	CTCTATGAACAAATTGAGGTGCAG	CGTTGATGATGGTAACTAGCTGAG
GmPHR5	ATTGAGGCTGAAGTTGTTTGTG	ATTCATCATCTGTAGCACTGCC
GmPHR6	CCAACTCAAGGGAAATCTGAC	TCTCAGTAATCAAGTGGCTATCAG
GmPHR7	AATGCTGGGGTTTTGGGAGGAT	TTTTGCAAGGCGATATTTCTGTAA
GmPHR8	CAGCAAACCAATCTGAAACC	GAGAAGAGAATCTCAGCATATGG
GmPHR9	CTAAGCGAAATGATTGGCAGGAGT	TTGATGGCTTTGAGATTGGTGTTC
GmPHR10	TTTGCCTTTAACTAACGGACAGAC	GTATTTCGGAAGACACGCTATCAC
GmPHR11	CATGATGCGTTTGGTTCTGTC	CAGTAACCTTGTTGCTTGTTGTC
GmPHR12	GAGCCACCTCCAGAAATTTAGG	CATGAAGTCTTCTCTGCACCT
GmPHR13	GGAGCTAGTAAAGCTACACCA	TTGGCAACTTTGGAGAAGTG
GmPHR14	CAGCGGCTCAGTGGCGTTCT	ATCTGGTGTCATCGGTTCAAGGTG
GmPHR15	AGCCAACTCAAGGGAAATCTG	GCCATTACTTGTCTTCTGCTG
GmPHR16	CTAGAGGTACAGAAGAAATTGCAG	TGATAATGGAAAGCTGAGTCGTG
GmPHR17	AATACCCCCAGAAGAGTTGTTT	ATTGGGTGATGTTAGTTGATGATT
GmPHR18	GTCTTGTTCTCACCTCTGATCC	CTGACATTCCATCTTTGAGTCC
GmPHR19	AACTTCAGGTGCAAAGACTC	GGCTAAATCCCTGTATCTCCT
GmPHR20	CCATCTCCAGAAATACAGATTGAG	CTGCAGAAGTCTTTGCACCT
GmPHR21	CTTGAGGTACAAAGACACTTGC	AATGCTGAGTTGAGGCACTG
GmPHR22	TCCCTTGCCAAAAATGAAATG	TCGGGGCTTGAGCACTATCT
GmPHR23	GAACAACTTGAGGTACAAAGACAC	TTGGTTTGTGGTTGCTGAGG
GmPHR24	GGAGCAAGTAAAGCTACACC	CCCTCATTTGTATCTGAAGTTGG
GmPHR25	AAAGGCCGACAAGAAAGAAACAGG	AACCACCGCTACAGCACCAGAAC
GmPHR26	GCCTTCAACTATGAATTCCCA	TGAATTTCTGAAGGTGGCTC
GmPHR27	GAAACACTGCTTCGTCTTCTG	CTCCTTCATCACTACTCCCA
GmPHR28	AAGAGTCATCTACAGAAGTACAGG	CTCAAGCTGCTCATGTAGTC
GmPHR29	TACGCTGGACACAAGAACTC	TCCTGTATTTCTGCAAATGGCT
GmPHR30	TGGGCCGCATAAATCTCAAG	GTCCAGGAACACCCATCACTCTAA
GmPHR31	CCAGTCACGGGAAATCTGAC	CCCTTCCTTGTTCCTCAATTCG
GmPHR32	GCGAAATGATTGGCAGGAGTGG	TTGGCAGGTGCAGAATTTGTTGAG
GmPHR33	AGGGGATCACTGAAGCATTACG	GTTTGTGTCCGAGGAGCCATTA
GmPHR34	ATTCCAGGACTCACTTTGTACCA	GGATTTCACTGTAACCTTCTTGCT
GmPHR35	GGGATGCAAAGATGGAATGTCAG	ATCTGTGCTCTTAATGCCTCC
GmPT1	CAGGTTCTGGCTAGGGTTTG	ACATAGTCAAATGCGGGGTC
GmPT2	GACATAGCGCGAAATCTGTC	CAAACACGGCCGCAATGAAG
GmPT3	ACAAGAAGACAAGAGGGTCG	AACCGAGCATGAGAATCAAC
GmPT4	AGGTGCACCAAAGCCGGGAACT	TGGCCATGACACCCTCTGCA
GmPT5	GAACACTTTCAGGGCAACTC	GTCATCACAGTCTTTGCATCG
GmPT6	CTGCTCACATACTATTGGCGT	GTCCAACAGGAACCAAGTAAC

Table S2 (continude)

Gene	Forward primer (5'-3')	Reverse primer (5'-3')
GmPT7	TGACCACAAGTACGATCTTCC	CGCCAATAGTAGGTAAGAGCA
GmPT8	TCATTTTCGCGGGTTTAGTC	GCTTGCTTCACGTTTCCTTC
GmPT9	ATGTTTAACTGTGGGCGGCG	CCCTATTATTGGGCGTCGGT
GmPT10	GGACTCCCGAATGAATGCTA	AGCTGCAGTCAACTCCCCTA
GmPT11	GAGCACTCCCAGCTGCATTG	GGCGACTGAGGAAGTCCTTG
GmPT12	GGACCACCAAGGAACATCATAA	CCCAGAAATGCCATGACAAC
GmPT13	GAGGGGCATTCATTGCTGCA	AGCGAATCCACCTTCGAACCT
GmPT14	GAGCAATTGGACACAAGAAG	TCCAACAGGAACCAAGTAGT
GmHAD1-2	TTGCACCCCAGAGTGATTCC	AGGGCATAAACTGCAGCCAT
GmSPX5	GATGCCAACGAACTCAACC	GAGCGAAGTAGAGCACCA
GmEXPB2	GAGGTCACCATCACCACTCTCAT	GGTGGTGCTTGTGGTTATGGAAGT
GmPAP14	CTCGGGGACAAGAAACAAAAGT	CAAACCAGATGGGGAGATGATAG
GmPAP21	GCTGATGGTGTTTGGATTG	TGTTGGGTGTCAAAGTTGAG
EF1-α	TGCAAAGGAGGCTGCTAACT	CAGCATCACCGTTCTTCAAA
ACTIN	ATCTTGACTGAGCGTGGTTATTC	GCTGGTCCTGGCTGTCTC

Table S3. Primer sequences used in this study for vector construction

Gene	Forward primer (5'-3')	Reverse primer (5'-3')
GFP-GmPHR25	GGGGACAAGTTTGTACAAAAAAGCAGGCTTCATGTATCATTCAAAGAATGTTCCTA	GGGGACCACTTTGTACAAGAAGCTGGGTCTCACAGATTACCGCCACC
GmPHR25-GFP	CGACGGTACCGCGGGCCCGGGATATGTATCATTCAAAGAATGTTCCTAG	TGGCGACCGGTGGATCCCGGGCCAGATTACCGCCACCAATC
OX-GmPHR25	GAGCTCATGTATCATTCAAAGAATGTTCCTAG	GACGTCTCACAGATTACCGCCACC
RNAi-GmPHR25(1)	CGGGATCCAACCTGATAATAAGACTGACCC	CCCAAGCTTCACCAATCTTAACCAATTCCTC
RNAi-GmPHR25(2)	CGACGCGTAACCTGATAATAAGACTGACCC	AACTGCAGCACCAATCTTAACCAATTCCTC
BD-GmPHR25	GAATTCATGTATCATTCAAAGAATGTTCCTAG	CTGCAGGTCACAGATTACCGCCACC
AD-GmPHR25	GCCATGGAGGCCAGTGAATTCATGTATCATTCAAAGAATGTTCCTA	ATGCCCACCCGGGTGGAATTCTCACAGATTACCGCCACC
GmPT9-pAbAi	CTTGAATTCGAGCTCGGTACCGTTGAAAGTTATAACACCGCTG	GTCGACAGATCCCCGGGTACCATCTCCCGTAGCATCATTCC
GmPT10-pAbAi	CTTGAATTCGAGCTCGGTACCATCTCCTTACATCTTCTAGCCT	GTCGACAGATCCCCGGGTACCTCAAATATCGACTGTTCAAGGG
GmPT12-pAbAi	CTTGAATTCGAGCTCGGTACCTTGAGCGCCATATAAGTGAG	GTCGACAGATCCCCGGGTACCGACGATCAAGGGAAGCAC

Table S4. Analysis of putative PHR1-binding site in a 3.0-kb sequence upstream of the start codon of each *GmPT* members

**Gene**	Putative P1BS element	Position
GmPT1	-	-
GmPT2	GTATATGC	-1845
GmPT3	-	-
GmPT4	-	-
GmPT5	GTATATGC	-1391
GmPT6	-	-
GmPT7	-	-
GmPT8	GAATATCC	-156
	GCATATCC	-187
GmPT9	GAATATCC	-159
	GCATATCC	-190
GmPT10	GAATATCC	-445
GmPT11	-	-
GmPT12	GTATATTC	-1171
	GTATATTC	-1395
GmPT13	-	-
GmPT14	-	-

Note, the position was given for the 5’-upstream nucleotide with respect to the first ATG in the transcribed region. The putative P1BS element upstream of transcriptional start site was analyzed by PLACE (http://www.dna.affrc.go.jp/PLACE/signalscan.html). - represents no P1BS element was detected.
